# Physiopathological correlations of comorbid insomnia and sleep apnoea (comisa) – a systematic review and meta-analysis

**DOI:** 10.1007/s11325-026-03631-0

**Published:** 2026-03-21

**Authors:** Ervin Cotrik, Janete Hernandes, Viviane Castro, Edilson Zancanella

**Affiliations:** 1 Postgraduate Program in Medical Sciences; Sleep Disorders Service of the Division of Otolaryngology, Head and Neck, University of Campinas - UNICAMP, São Paulo, Brasil; 2 Department of Sleep Medicine Research, Instituto de Pesquisa Capel Castro, Goiânia, Goiás Brasil

**Keywords:** COMISA, Insomnia, Obstructive sleep apnoea, Physiopathology

## Abstract

**Purpose:**

To ascertain the physiopathological correlations of insomnia and obstructive sleep apnoea (Comorbid Insomnia and Sleep Apnoea (COMISA).

**Methods:**

Systematic review and meta-analysis, with searches in PubMed, Embase, Web of Science, Scopus, Cochrane, and grey literature, including physiopathological characteristics and prevalence of COMISA in observational studies. The meta-analysis employed the I² and Cochran’s Q tests, as well as the random effects method, and the results were presented in forest plots. The risk of bias was assessed using the Joanna Briggs Institute Critical Appraisal tools.

**Results:**

Sixteen studies were selected, with a prevalence of 30% for COMISA, 39% among ages 18–40, 42% in European countries, and an average Body Mass Index (BMI) of 28.55 (± 1.12). Patients with COMISA have higher Apnoea-Hypopnea Index (AHI), lower minimum oxygen saturation, higher micro-arousal index, longer wake time after sleep onset Wake Time After Sleep Onset (WASO), lower sleep efficiency, longer sleep latency, and possibly shorter Rapid Eye Movement (REM) sleep duration.

**Conclusion:**

The physiopathology of COMISA involves compromised sleep architecture and a complex interaction of mechanisms related to central arousal, sleep fragmentation, neurobiological changes, and a more severe profile of clinical and psychiatric comorbidities, with additive or synergistic consequences of the isolated disorders, requiring a comprehensive and personalized diagnostic and therapeutic approach.

**PROSPERO Registry number**: CRD420251038279.

**Supplementary Information:**

The online version contains supplementary material available at 10.1007/s11325-026-03631-0.

## Introduction

Sleep disorders encompass a broad spectrum of pathologies that significantly impair quality of life and overall well-being. Among the sleep-related dysfunctions with the greatest epidemiological relevance, insomnia and obstructive sleep apnoea syndrome (OSA) stand out. The co-occurrence of these two prevalent disorders forms a complex clinical entity known as COMISA (comorbid insomnia and obstructive sleep apnoea) [1].

In this context, current data indicate that chronic insomnia has a prevalence ranging from 10% to 40% of the population, increasing the risk of psychiatric disorders, deteriorating quality of life, and reducing work productivity, thereby constituting a major public health concern. Insomnia is characterized by persistent difficulties in initiating or maintaining sleep, accompanied by significant impairments in daytime functioning. When these symptoms occur at least three nights per week for a period of three months or longer, and are associated with substantial distress or functional impairment, the diagnosis of chronic insomnia is established [2–4].

Similarly, OSA is defined by the recurrent occurrence of partial or complete collapses of the upper airway during sleep, leading to oxygen desaturation, micro-arousals, and sleep fragmentation. Isolated sleep apnoea is characterized by an apnoea-hypopnea index (AHI) ≥ 5 events per hour on overnight polysomnography, with a reduction in nasal airflow amplitude greater than 90% and lasting at least 10 s per event. The prevalence of OSA is also notably high, affecting between 9% and 38% of the adult population [5, 6].

Advancing in conceptualization, the concept of COMISA was first described by Guilleminault and colleagues in 1973; however, it has only gained progressive recognition within the global scientific community over the past two decades. COMISA is a condition characterized by bidirectional pathophysiological interactions. In contrast to earlier terminology, the term COMISA was introduced to unify nomenclature and accurately convey the comorbid nature of this clinical entity [1, 7]. Initially, insomnia was considered secondary to OSA; however, recent evidence indicates a bidirectional and causal relationship between the two conditions, whereby each disorder can trigger, perpetuate, or exacerbate the other [8]. Recent epidemiological studies have reported a significant increase in COMISA prevalence within the general population, reaching 22.95% after an eight-year follow-up in longitudinal research [9]. It is noteworthy that COMISA prevalence is even higher in certain specific populations, such as military personnel (38.2–88%) and trauma victims (52%) [10].

Current evidence shows that approximately 30–40% of patients with OSA exhibit significant insomnia symptoms, while 30–50% of individuals with chronic insomnia meet diagnostic criteria for OSA [11]. The pathophysiology of COMISA involves complex and interconnected mechanisms. A reduced arousal threshold and hyperexcitability may predispose individuals to this comorbid condition [12]. Repetitive awakenings triggered by OSA can lead to the development of maintenance insomnia, whereas insomnia – through increased cortical excitability and frequent awakenings – may exacerbate ventilatory instability and contribute to the pathogenesis of OSA [13]. This bidirectional relationship creates a vicious cycle that perpetuates both conditions.

Additionally, the clinical manifestations of COMISA differ significantly from those observed in isolated disorders. Individuals with COMISA experience greater sleep disruption, poorer quality of life, unfavorable functional outcomes, and a higher prevalence of psychiatric comorbidities [14]. Objectively, these patients exhibit reduced total sleep time, increased sleep onset latency, decreased sleep efficiency, and prolonged wake time after sleep onset [15].

This systematic review and meta-analysis addresses the increasing prevalence of COMISA and the associated challenges in its clinical management, aiming to clarify its specific pathophysiological correlations. Despite the recognized importance of this condition, a significant gap persists in the scientific understanding of its specific pathophysiological correlations.

The novelty of this study resides in its systematic and comprehensive approach to the pathophysiological interactions between comorbid insomnia and sleep apnoea, offering a critical synthesis of the current literature and identifying future directions for research and clinical practice within this key domain of sleep medicine.

The objective of this systematic review was to examine the pathophysiological correlation of insomnia and obstructive sleep apnoea (COMISA). The specific aims were to identify the clinical characteristics of patients with COMISA; to determine the prevalence of COMISA in the general population by age, geographic region, and Body Mass Index (BMI); and to compare polysomnographic indicators among patients with COMISA, isolated sleep apnoea, and isolated insomnia.

## Method

Systematic review, with its protocol registered on the prospective registry site CRD420251038279. Structured in accordance with the guidelines of the Preferred Reporting Items for Systematic Reviews and Meta-Analyses (PRISMA) 2020 checklist.

### Eligibility criteria

We selected studies according to the following eligibility criteria: patients of any age, regardless of sex, from any geographic region; diagnosed with insomnia and obstructive sleep apnoea (COMISA); with or without a comparison group; reporting outcomes related to COMISA characteristics, prevalence, and associations; any study design, with no restrictions on publication date or language. Literature reviews, letters, editorials, protocols, and conference abstracts were excluded.

### Sources of information

We searched PubMed, Embase, Web of Science, Scopus, and Cochrane. The Brazilian Digital Library of Theses and Dissertations (BDTD) and the artificial intelligence tool Research Rabbit were employed for gray literature. Database searches were conducted in January 2025, with no language restrictions applied. The complete list of terms used in each database is available in Table 1 (Supplementary Material).

### Procedures and data collection

We selected keywords and search terms using Health Sciences Descriptors (DeCS), Medical Subject Headings (MeSH), and Emtree. The following terms were included: COMISA OR Co-morbid insomnia and sleep apnoea OR Comorbid insomnia and sleep apnoea AND pathophysiology. Three independent reviewers participated in all stages and phases of the systematic review process.

We conducted the review in three phases. In the first phase, articles were retrieved from the databases (Identification). In the second phase, duplicate articles were identified, and inclusion and exclusion criteria were applied through screening of titles and abstracts (Screening). In the third phase, data were extracted from the selected studies (Inclusion). All these phases are illustrated in a flow diagram presented in the Results section of this study.

### Data analysis

After a full-text review of the articles, we confirmed the feasibility of conducting a meta-analysis. We assessed heterogeneity using the I² statistic based on Cochran’s Q test. We presented the results in forest plots. We employed Comprehensive Meta-Analysis (Version 4.0.000) and Cochrane’s Review Manager (RevMan, Version 5.4.1) for the analyses. A p-value < 0.05 indicated statistical significance for all tests. We performed an analysis of the strength of evidence using the GRADE approach (The Grading of Recommendations Assessment, Development and Evaluation).

### Risk of bias analysis

To assess the risk of bias in the selected studies, we applied the Joanna Briggs Institute tools for observational studies. We considered studies that met 80% of the items as ‘high’ quality with a low risk of bias. We classified those meeting between 60% and 79% of the items as ‘moderate’ quality with a moderate risk of bias, while we regarded studies meeting fewer than 59% of the items as ‘low’ quality with a high risk of bias.

## Results

Initially, 249 records were identified in the databases. Thirty-eight duplicates were excluded, leaving 211 for the next phase. During the title screening, 110 records were excluded, and 81 were excluded during the abstract screening for not meeting the inclusion criteria, resulting in 20 studies. After full-text review, 4 studies were excluded, leaving 16 studies for the systematic review and meta-analysis. No records were found through manual search.

**Fig. 1:**
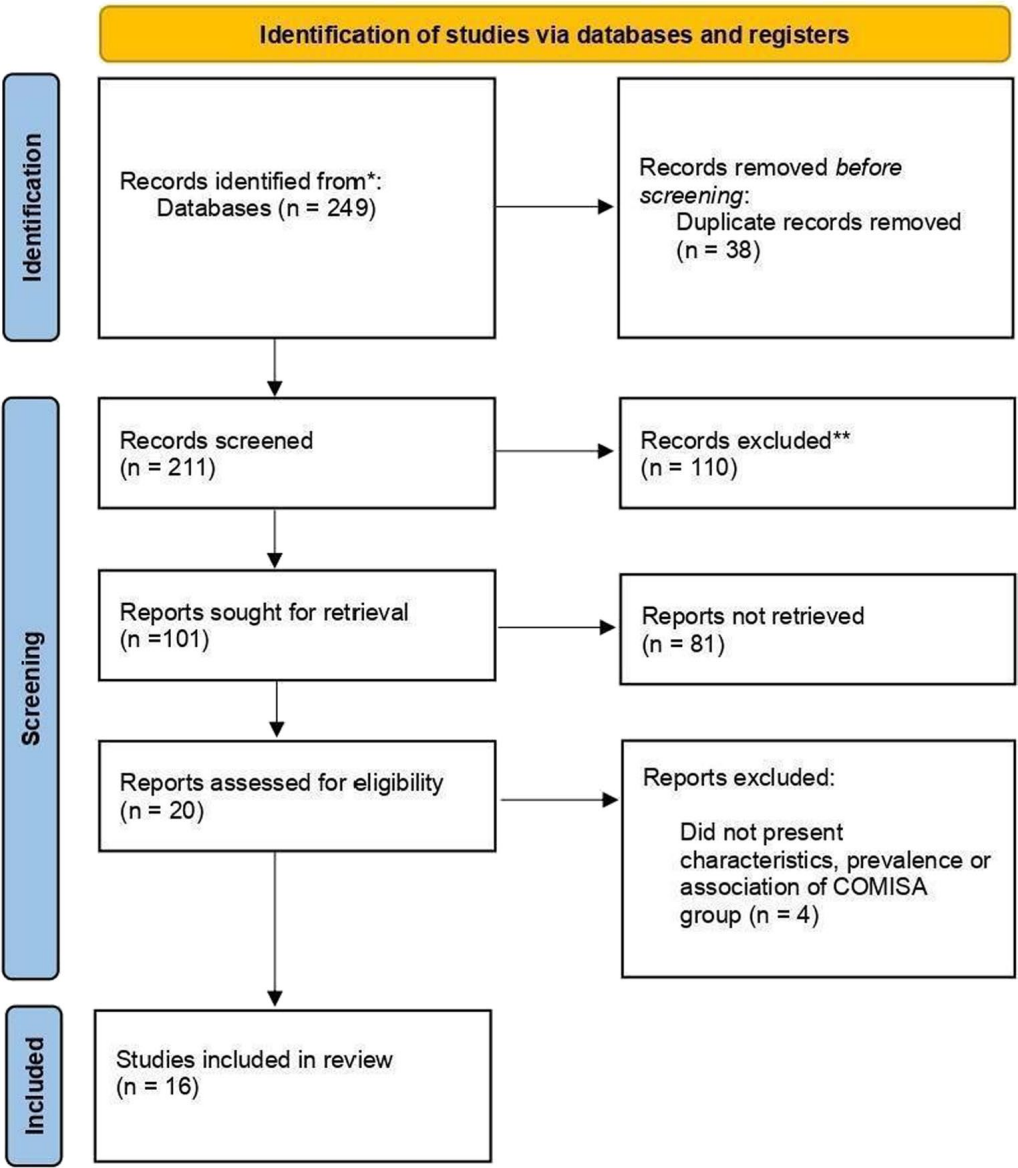
Flow diagram (PRISMA, 2020)

Therefore, 16 studies were selected for qualitative data synthesis, as shown in Fig. 1 and Supplementary Material 1.

Examining the study characteristics, the selected studies included more than 9,700 participants across a total of sixteen observational studies. The mean age of participants was 42.56 years. Based on five studies [16–20] that reported sample size (N), mean BMI, and Standard Deviation (SD), the combined weighted mean BMI was approximately 30.79 kg/m², with a combined standard deviation of approximately 6.79 kg/m². The studies were conducted in various countries, including Portugal, Brazil, the United States, Iran, India, China, Mexico, the Netherlands, Israel, Norway, South Korea, Iceland, and Australia, as shown in Supplementary Material 2.

The assessment of Obstructive Sleep Apnoea and Insomnia (COMISA), sleepiness, fatigue, mood disorders, depression, anxiety, sleep, breathing, and gray matter was performed using instruments based on the criteria of the International Classification of Sleep Disorders (ICSD-2/3) and/or DSM-IV, as shown in Supplementary Material 3.

### Risk of bias analysis

As shown in Supplementary Material 4, eight studies were classified as having a low risk of bias and, consequently, high quality, while eight studies were evaluated as having a moderate risk of bias, indicating moderate quality. For cross-sectional studies, the items with lower ratings were mainly related to question 5 and question 6. In the case-control study, the items with lower ratings were question 1, question 2 and question 3.

### Meta-analysis

For the construction of the meta-analysis, COMISA event frequencies were used to determine prevalence in the studied general population, age group, geographic region, and BMI. The random-effects model was applied to all data analyses due to the high heterogeneity among studies.

**Fig. 2:**
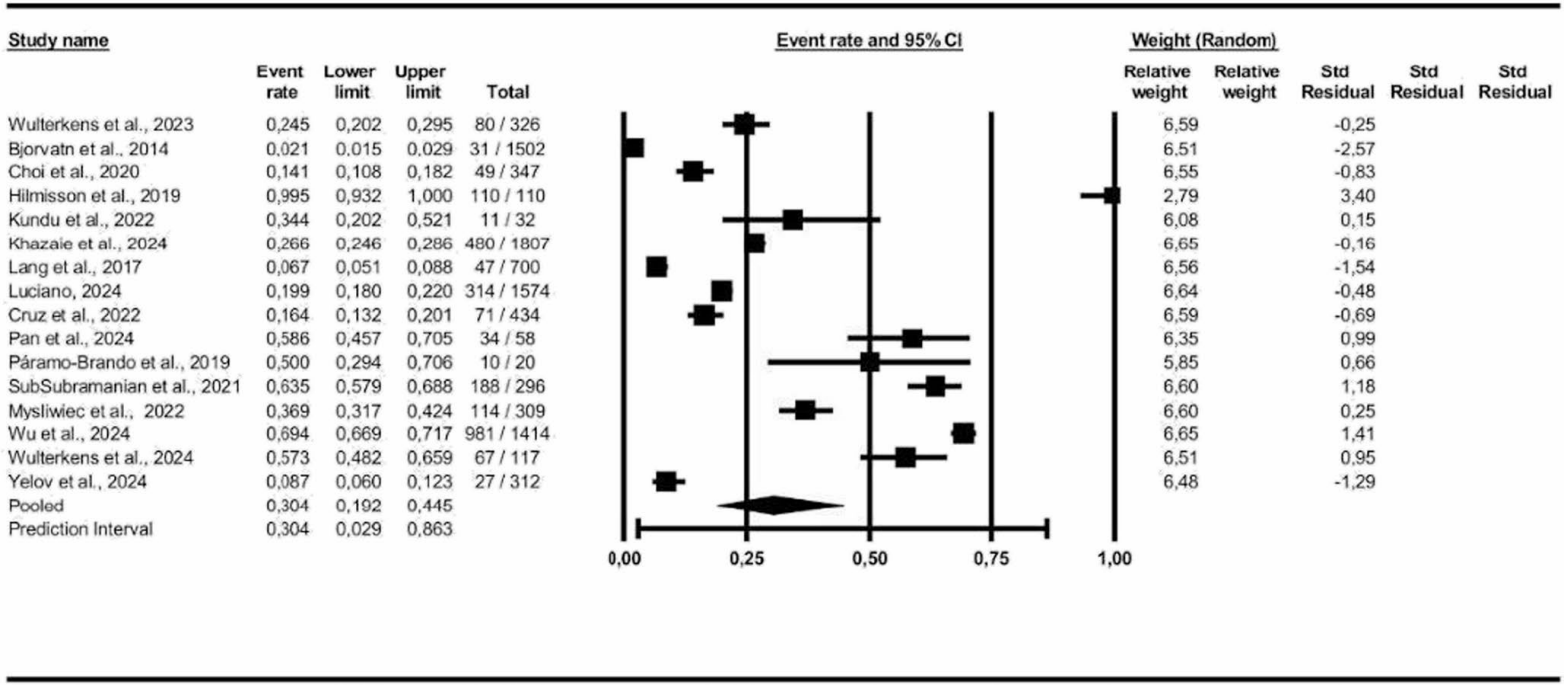
COMISA Prevalence among participants in the selected studies

For the first analysis (COMISA prevalence), all 16 studies were included, and the results indicate a prevalence of 30% for COMISA in the studied population (95% Confidence Interval (CI): 0.19 to 0.44, *p* < 0.05; I² = 99%), as illustrated in Fig. 2.

**Fig. 3:**
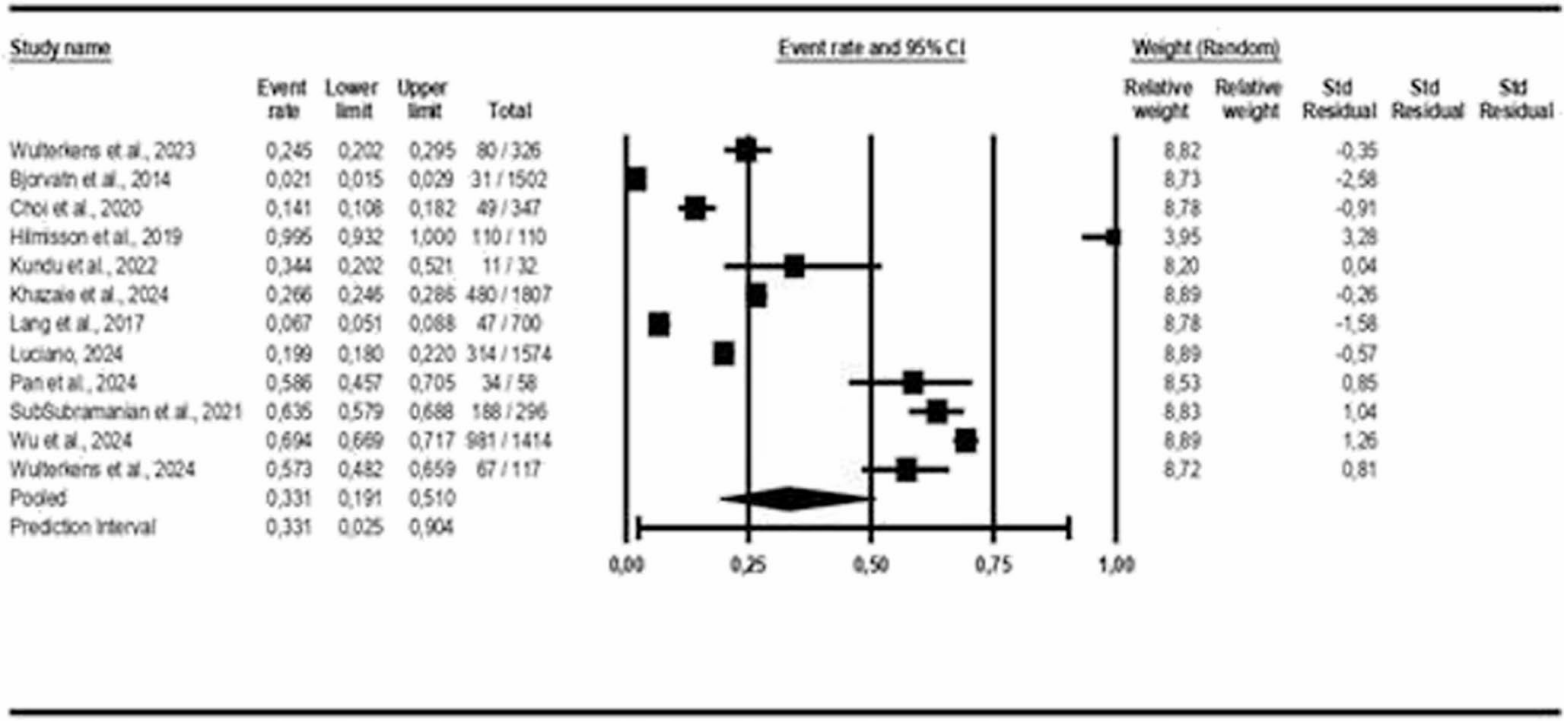
Prevalence of COMISA among participants over 40 years

**Fig. 4:**
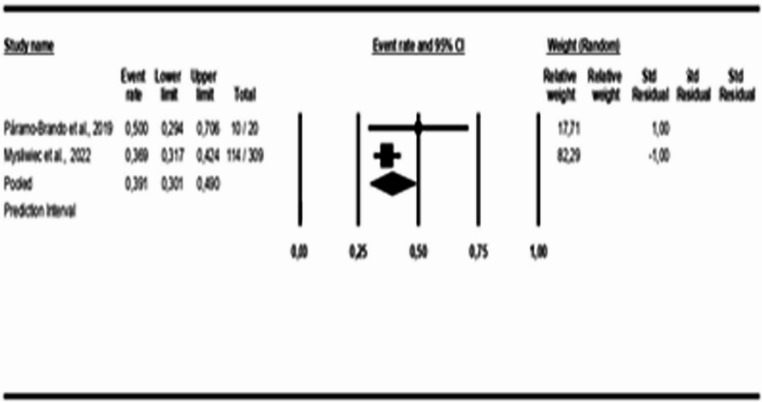
Prevalence of COMISA among participants aged 18 to 40 years

**Fig. 5:**
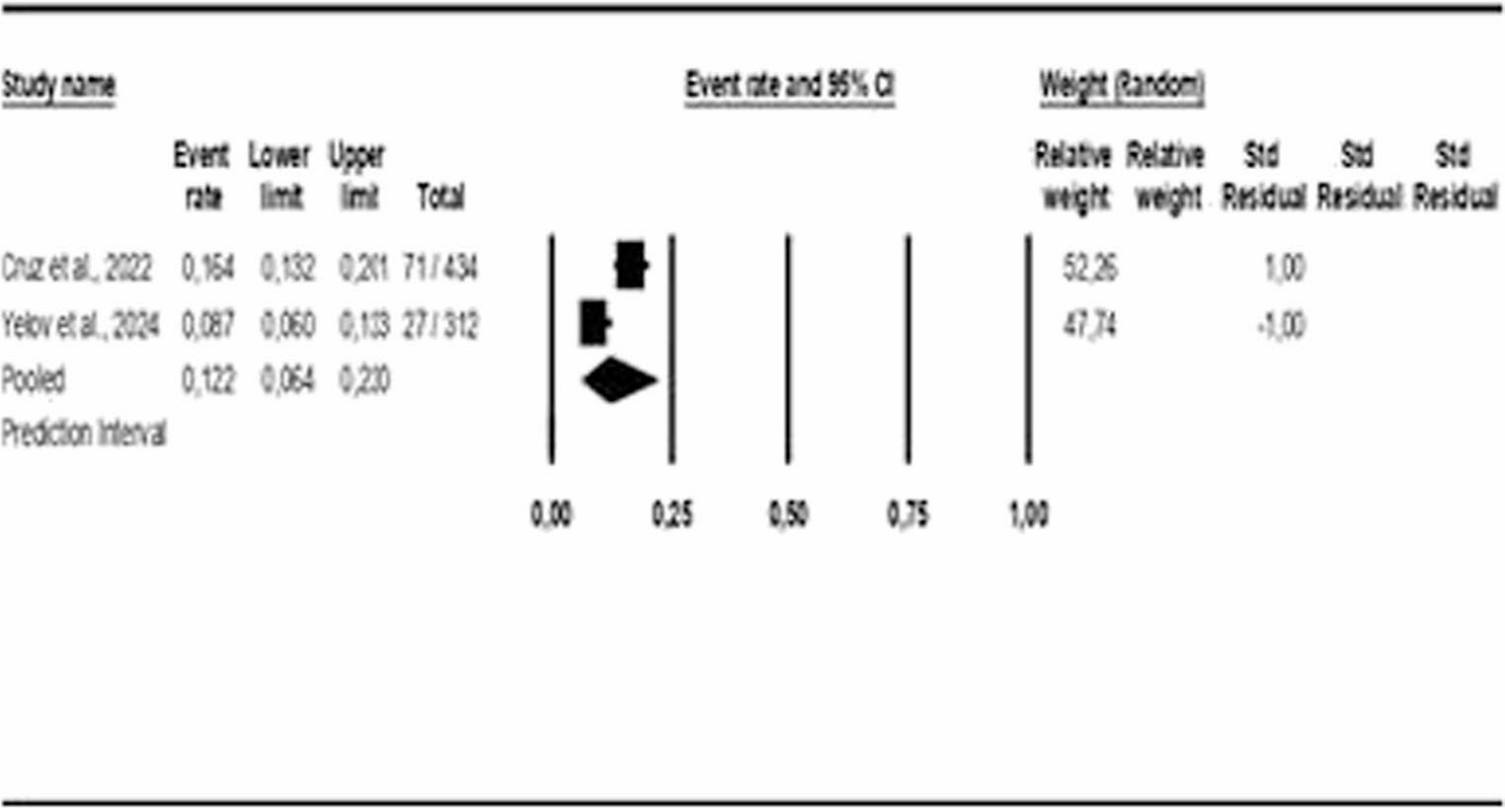
Prevalence of COMISA among children and adolescent participants

For the age-group analysis, three subgroups were created: mean age above 40 years [9, 16, 18, 21–28], mean age between 18 and 40 years [17, 19], and children and adolescents [29]. The results indicate a COMISA prevalence of 33% in the population over 40 years (95% CI: 0.19 to 0.51, *p* < 0.05; I² = 99%), 39% in the population aged 18 to 40 years (95% CI: 0.30 to 0.49, *p* > 0.05; I² = 26%), and 12% in the population of children and adolescents (95% CI: 0.06 to 0.22, *p* < 0.05; I² = 89%), as shown in Figs. 3 and 4, and 5.

**Fig. 6:**
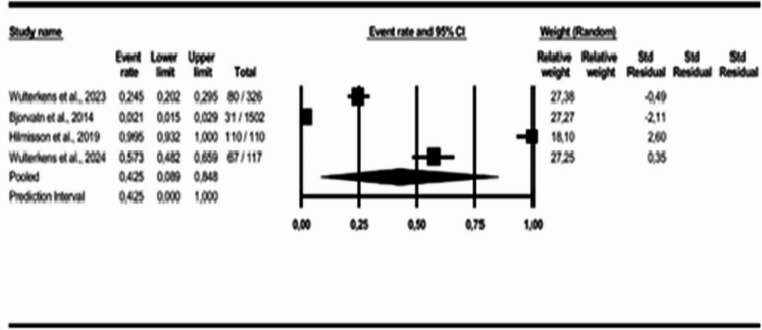
Prevalence of COMISA in European countries

**Fig. 7:**
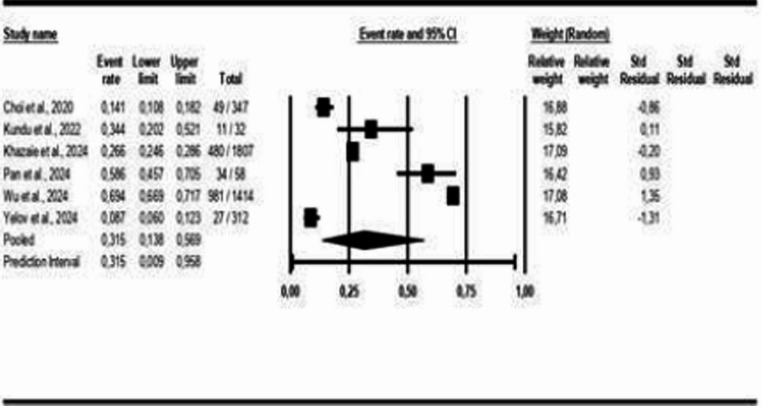
Prevalence of COMISA in Asian countries

**Fig. 8:**
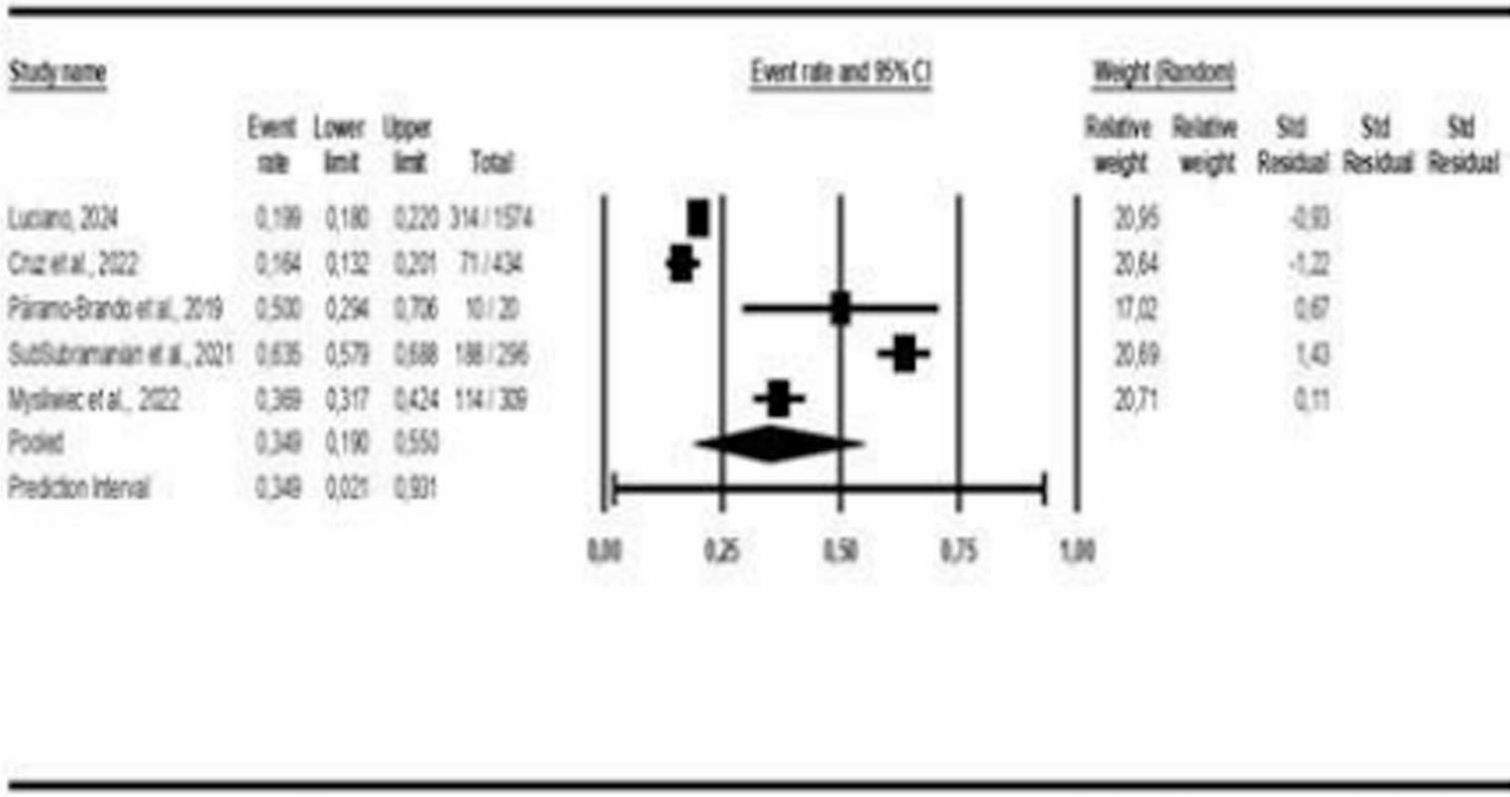
Prevalence of COMISA in American countries

For the geographic region analysis, three subgroups were created: European countries [20–22, 28], Asian countries [16, 18, 23, 24, 27, 29], and American countries [9, 17, 19, 26]. The results indicate a COMISA prevalence of 42% in European countries (95% CI: 0.09 to 0.85, *p* < 0.05; I² = 99%); 31% in Asian countries (95% CI: 0.14 to 0.57, *p* < 0.05; I² = 99%); and 35% in American countries (95% CI: 0.19 to 0.55, *p* < 0.05; I² = 98%), as shown in Figs. 6 and 7, and 8.

**Fig. 9:**
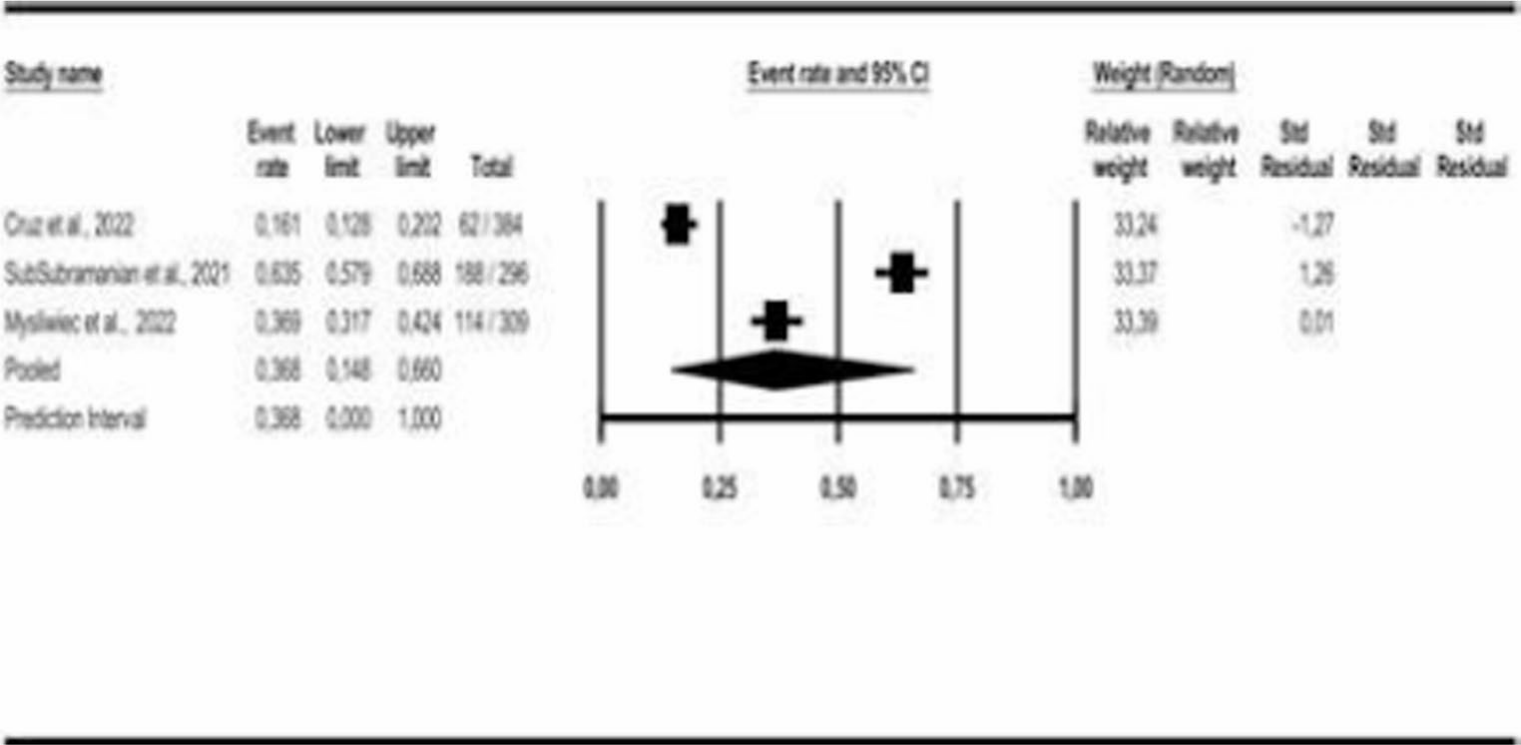
Prevalence of COMISA in the USA

**Fig. 10:**
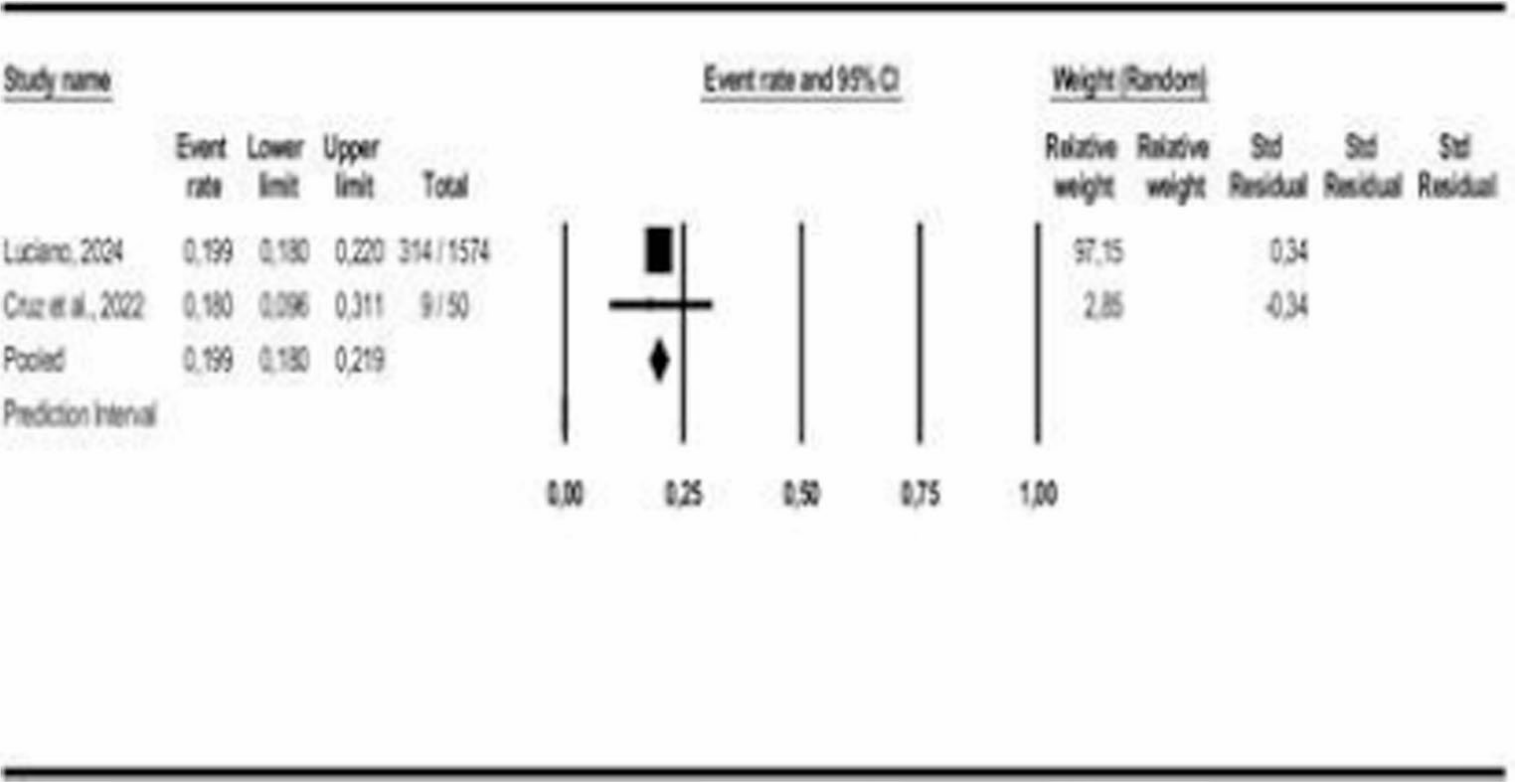
Prevalence of COMISA in Brazil

An analysis of COMISA prevalence was conducted specifically for Brazil [2, 9] and the United States [17, 26]. The results indicate a prevalence of 37% for COMISA in the United States (95% CI: 0.15 to 0.67, *p* < 0.05; I² = 99%) and 20% in Brazil (95% CI: 0.18 to 0.22, *p* > 0.05; I² = 0%), as shown in Figs. 9 and 10.

**Fig. 11:**
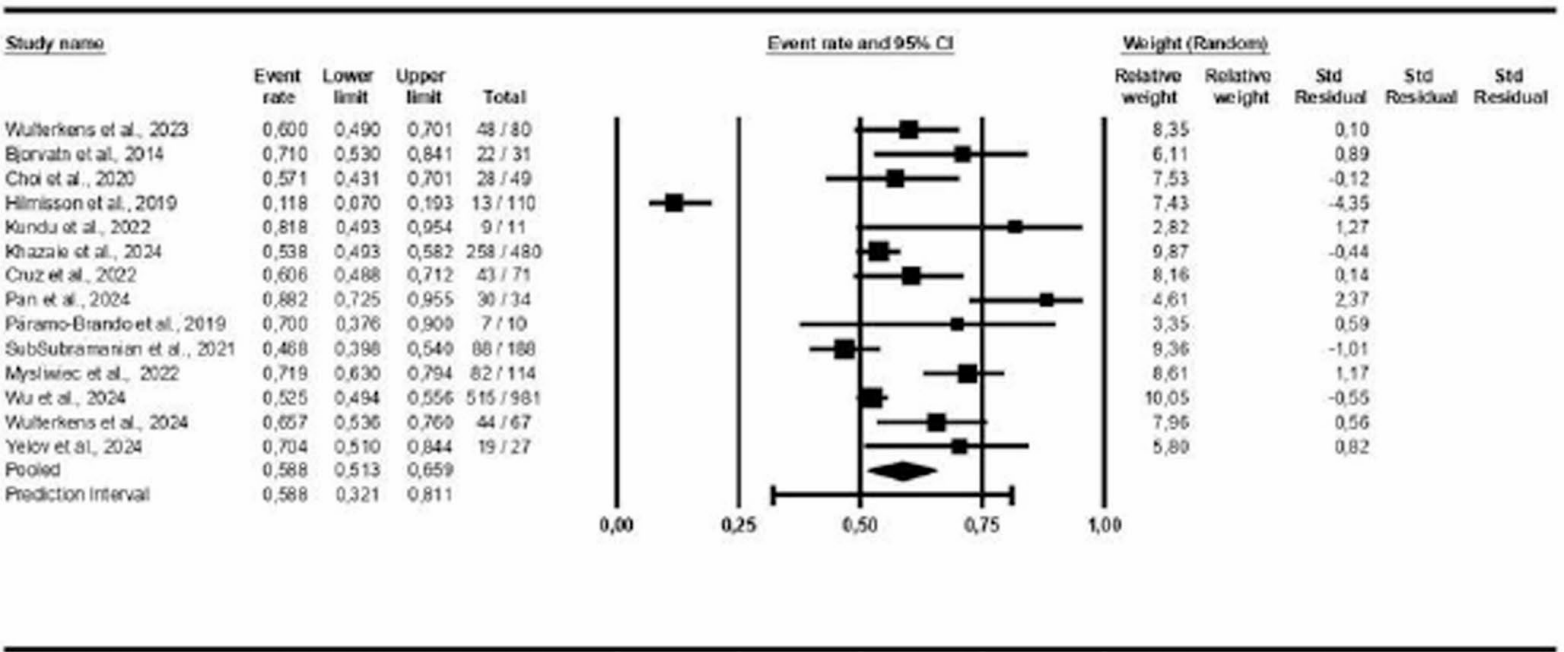
Prevalence of COMISA in men

**Fig. 12:**
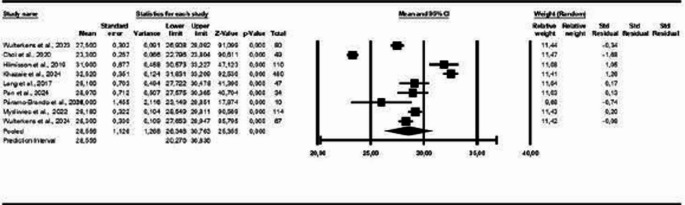
Average BMI in COMISA

In the sex-based analysis, the results indicate a prevalence of 59% of COMISA in the male population (95% CI: 0.51–0.66, *p* < 0.05; I² = 87%), as shown in Fig. 11. In the analysis by Body Mass Index (BMI), the findings indicated an average BMI of 28.55 (± 1.12) among participants with COMISA (95% CI: 26.34 to 30.76; *p* < 0.05; I² = 98%), as illustrated in Fig. 12.

For the meta-analysis involving polysomnograpy indicators, 09 studies were used [16, 17, 19, 21, 23, 24, 27–29]. To allow standardized comparative analysis, all variables originally described as Median (P25-P75) were converted to the format Mean (± estimated SD). The transformation was carried out based on the valid approximation for symmetric distributions, where: SD ≈ (P75 - P25)/1.35. This factor (1.35) represents the typical width of the interquartile range in a standard normal distribution, as shown in Supplementary Material 5.

The studies by Pan et al., 2024 [18], Subramanian et al., 2021 [26], Bjorvatn 2014 [22], Hilmisson 2019 [20], Luciano 2024 [9], and Lang (2017) [25] were excluded for not presenting results in accordance with the criteria established for this meta-analysis.

**Fig. 13:**
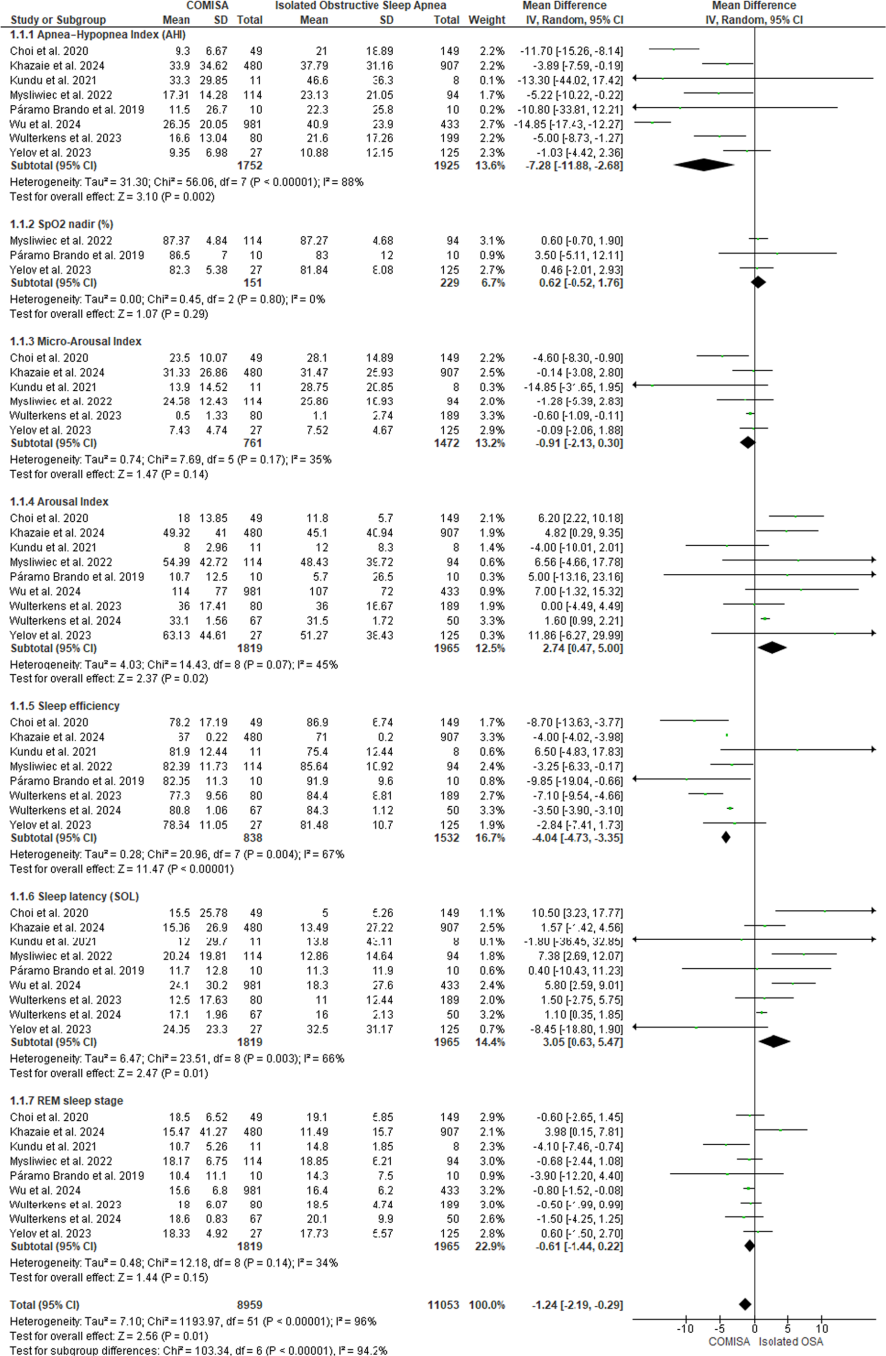
Polysomnography Indicators in patients with COMISA and isolated Obstructive Sleep Apnoea (OSA)

Figure 13 displays the studies that presented polysomnography indicators in patients with COMISA and isolated OSA. The results show that there is a small statistically significant difference between the COMISA and OSA groups, MD = −1.24 (95% CI: −2.19 to −0.29, *p* < 0.05, I² = 94%), which can also be observed in the following polysomnography indicators: AHI: MD = −7.28 (95% CI: −11.88 to −2.68, *p* < 0.05, I² = 88%); Arousal Index: MD = 2.74 (95% CI: 0.47 to 5.00, *p* < 0.05, I² = 45%), Sleep Efficiency: MD = −4.04 (95% CI: −4.73 to −3.35, *p* < 0.05, I² = 67%) and Sleep Latency: MD = 3.05 (95% CI: 0.63 to 5.47, *p* < 0.05, I² = 66%), except for SpO₂: MD = 0.62 (95% CI: −0.52 to 1.76, *p* > 0.05, I² = 0%); Microarousal Index: MD = −0.91 (95% CI: −2.13 to 0.30, *p* > 0.05, I² = 35%) and REM Sleep Duration: MD = −0.61 (95% CI: −1.44 to 0.22, *p* > 0.05, I² = 34%), which did not show statistically significant differences between the groups. For the Arousal Index and Sleep Latency, the higher means are on the COMISA group.

**Fig. 14:**
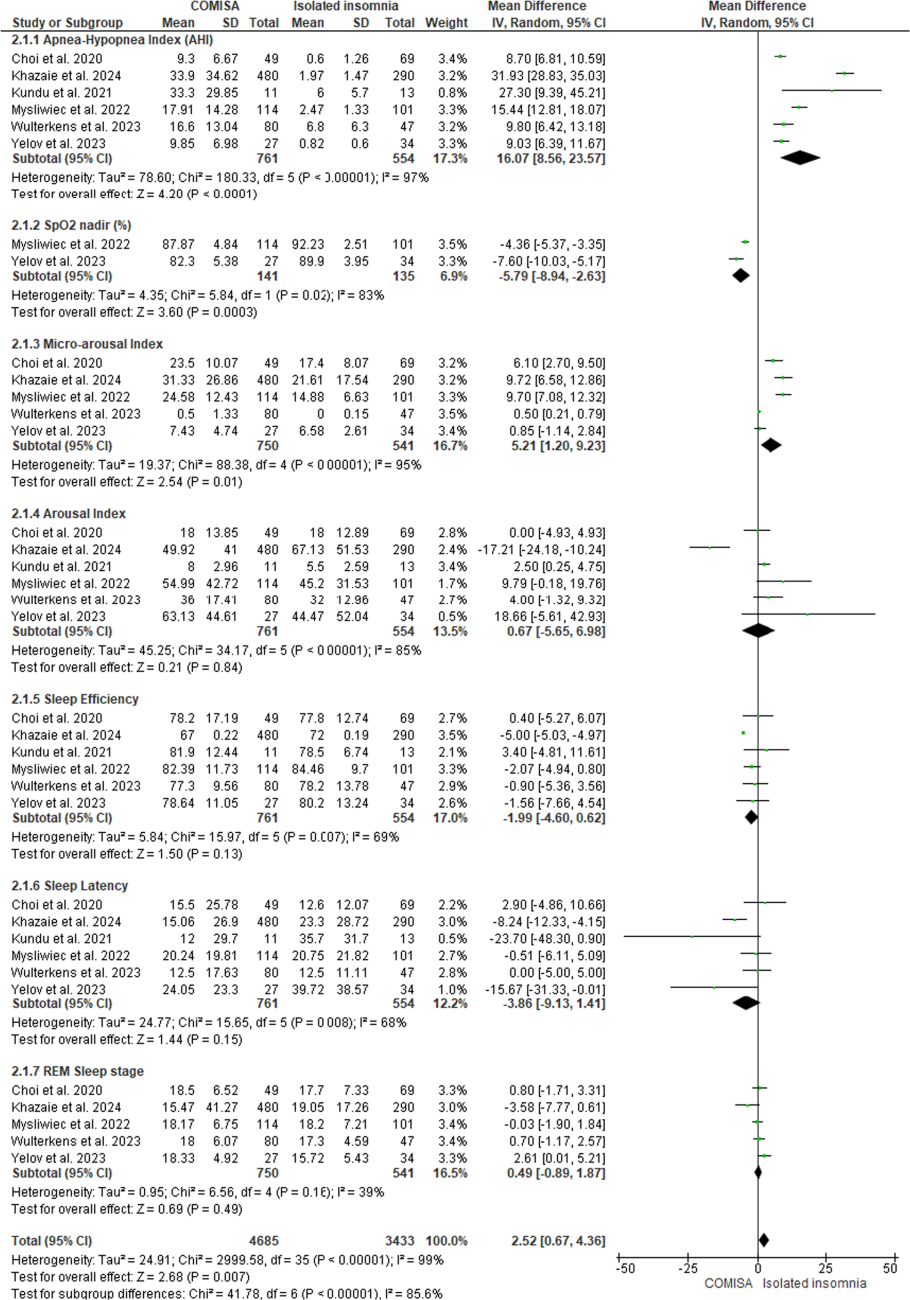
Polysomnography Indicators in patients with COMISA and isolated Insomnia

Figure 14 shows the studies that presented polysomnography indicators in patients with COMISA and isolated insomnia. The results show that there is a small statistically significant difference between groups: MD = 2.52 (95% CI: 0.67 to 4.36, *p* < 0.05, I² = 99%), which can also be observed in the following polysomnography indicators: AHI: MD = 16.07 (95% CI: 8.56 to 23.57, *p* < 0.05, I² = 97%), Minimum SpO₂: MD = −5.79 (95% CI: −8.94 to −2.63, *p* < 0.05, I² = 83%), Microarousal Index: MD = 5.21 (95% CI: 1.20 to 9.23, *p* < 0.05, I² = 95%), except for Arousal Index: MD = 0.67 (95% CI: −5.65 to 6.98, *p* > 0.05, I² = 85%); Sleep Efficiency: MD = −1.99 (95% CI: −4.60 to 0.62, *p* > 0.05, I² = 69%); Sleep Latency: MD = −3.86 (95% CI: −9.13 to 1.41, *p* > 0.05, I² = 68%) and REM Sleep Duration: MD = 0.49 (95% CI: −0.89 to 1.87, *p* > 0.05, I² = 39%), which did not show statistically significant differences between the groups. For the polysomnography indicators AHI and Microarousal Index, the higher means are on the COMISA group.

#### The grading of recommendations assessment, development and evaluation - GRADE

The strength of the evidence for the analysis conducted between COMISA and isolated Obstructive Sleep Apnoea was considered high for the polysomnography indicators AHI and Arousal Index. For the other indicators, it was considered Moderate, as shown in Supplementary Material 6.

The strength of the evidence for the analysis conducted between COMISA and isolated Insomnia was considered high for the polysomnography indicator AHI. For the Microarousal Index and REM Sleep Duration indicators, it was considered Moderate. As for the other indicators, it was considered low as shown in Supplementary Material 7.

## Discussion

The combination of characteristics of OSA with comorbid insomnia (COMISA) results in a deterioration of sleep architecture, particularly when compared to isolated comorbid insomnia [9, 17, 23, 27].

In general, when analyzing the polysomnography data, we observe small differences among COMISA, isolated OSA, and isolated comorbid Insomnia, with particular emphasis on AHI. Although the macroarchitecture of sleep (sleep stages, total time) may appear similar between groups in Polysomnography (PSG), the Apnoea-Hypopnea Index (AHI) emerges as the main clinical and pathophysiological indicator for differentiating these groups. For patients with COMISA, AHI represents the OSA component that interacts bidirectionally with insomnia, potentially exacerbating it and leading to frequent awakenings following obstructive events. OSA worsens insomnia because respiratory events (measured by the AHI) force the brain to wake up to resume breathing, which leads to the development of maintenance insomnia, and insomnia worsens OSA because the state of cortical hyperalertness and sleep fragmentation typical of insomnia can lower the arousal threshold (the patient wakes up before even completing an effective respiratory maneuver), which increases ventilatory instability and, consequently, can increase the number of events recorded in the AHI [21, 24].

As observed, the relationship between OSA and insomnia is bidirectional, meaning that the symptoms of one condition can exacerbate or predispose to the other, resulting in a more severe sleep phenotype and a worse overall prognosis for COMISA. This leads to shorter Total Sleep Time (TST), lower Sleep Efficiency (SE), longer Sleep Onset Latency (SOL), frequent nocturnal awakenings due to obstructive events or nocturia, and lighter sleep compared to patients who have only OSA or isolated insomnia [9, 28, 30].

Sleep fragmentation induced by OSA, combined with the low arousal threshold characteristic of insomnia, can lead to prolonged and more variable awakenings throughout the night in patients with COMISA; a finding that may not be as evident in single-night polysomnography but becomes clearer in multi-night measurements [21, 28].

In patients with COMISA, there is a greater average duration of awakenings and a higher number of awakenings lasting 5 min or more (WKN ≥ 5 min), resulting in an extended WASO that includes only these longer awakenings (WASO ≥ 5 min), compared to patients with OSA alone [22, 29]. Changes in sleep stages are also observed, such as an increase in N1/N2 sleep at the expense of N3 [23, 27].

Sleep state misperception (SSM) is common in patients with COMISA, contributing to the subjective perception of poor sleep quality even when objective measures may be less severe [24, 25, 29].

The discrepancy between objective polysomnography findings and subjective reports of poor sleep in COMISA suggests a qualitative impairment of sleep microstructure not captured by conventional metrics. Studies indicate that the mistaken perception of sleep state may be related to alterations in slow-wave activity and an increase in the power of fast frequencies (beta and gamma) during NREM sleep. In COMISA, this intrusion of wakefulness into sleep may be amplified by intermittent hypoxia, which promotes an altered pattern of functional brain connectivity, resulting in sleep that, although present in the recordings, is processed by the patient as an unsatisfactory and fragmented state of wakefulness [9, 19, 23, 24].

The meta-analysis on overall COMISA prevalence is consistent with the data presented by Luciano et al. [9]. COMISA prevalence generally increases with age [16, 27]. However, in children, the presentation may differ; for example, daytime sleepiness may be less prominent, and sleep problems may manifest as hyperactive daytime behavior [29].

Furthermore, the meta-analysis by geographic region revealed lower prevalence rates in Brazil. This may be related to the fact that the study by Cruz et al., 2022, included in the meta-analysis, was conducted with children and adolescents, populations with lower COMISA prevalence compared to other groups. The highest prevalence of COMISA patients was observed in European countries, aligning with the findings of Zhang et al. [11], where the highest prevalence rates were found in European and American countries.

The prevalence of Comorbid Insomnia and OSA (COMISA) shows significant regional variations, reaching a higher percentage in European countries. These data demonstrate that the distribution of the condition is influenced by geographic location, which may reflect ethnic and genetic disparities, as well as sociocultural and environmental variations in the perception of insomnia [2, 16, 24]. Although the results were based on adjusted statistical models, it is possible that these factors are influencing the results, which is a limitation of the study [2, 16, 24].

The weighted mean BMI of 30.79 kg/m² in our sample indicates a predominantly obese population. While obesity is a primary driver of OSA through mechanical airway collapse, it can also contribute to central sleep apnoea and obesity hypoventilation syndrome, potentially confounding the AHI results. Future studies should stratify COMISA patients by BMI to isolate the specific neurobiological interactions from obesity-related mechanical effects [9, 18, 22, 24].

From a clinical standpoint, identifying specific pathophysiological phenotypes in COMISA is crucial to overcoming the historically low rates of CPAP adherence. The integration of Cognitive-Behavioral Therapy for Insomnia (CBT-I) before or concurrently with the initiation of apnoea treatment should not be seen merely as an adjunct treatment, but as a necessary pathophysiological intervention to stabilize the arousal threshold and reduce hyperarousal, facilitating acceptance of positive pressure therapy. This reinforces the need for a personalized and integrated therapeutic approach, such as the combination of Cognitive Behavioral Therapy for Insomnia (CBT-I) and continuous positive airway pressure (CPAP), since treating only one component may not be sufficient to mitigate the overall health risks to the patient [8, 13, 16, 17, 21, 26].

The inclusion of the studies by Mysliwiec et al. [17] and Khazaie et al. [16] constitutes a limitation of this review due to the presence of shift workers in their samples, which introduces significant confounding variables into the COMISA analysis. Shift work is a known factor in precipitating and perpetuating sleep disorders through circadian misalignment and chronic sleep deprivation, elements that can artificially mimic or exacerbate insomnia symptoms and the severity of obstructive sleep apnoea.

New studies are needed to compare the main polysomnographic indicators and verify whether the results remain consistent regarding the COMISA, isolated Apnoea, and isolated Insomnia groups.

## Conclusion

The pathophysiology of COMISA goes beyond the mere sum of two disorders, requiring a comprehensive and personalized diagnostic and therapeutic approach.

The objective of this systematic review was to examine the pathophysiological correlations of insomnia and obstructive sleep apnoea (COMISA). Differences are particularly evident in the indicators AHI, Minimum SpO₂, Microarousal Index, Arousal Index, Sleep Efficiency, and Sleep Latency, which show greater impairment in patient groups with COMISA. Prevalence remains very similar to that already reported in the literature, around 30% of the general population studied.

## Supplementary Information

Below is the link to the electronic supplementary material.

Supplementary Material 1 (DOCX 1.73 MB)

Supplementary Material 2 (DOCX 2.58 MB)

Supplementary Material 3 (DOCX 1.74 MB)

Supplementary Material 4 (DOCX 1.74 MB)

Supplementary Material 5 (DOCX 1.74 MB)

Supplementary Material 6 (DOCX 2.61 MB)

Supplementary Material 7 (DOCX 2.61 MB)

## Data Availability

The data supporting the results of this study are available from the corresponding author upon reasonable request at cotrikpsiquiatria@gmail.com.

## Abbreviations

AHI: Apnoea-Hypopnea Index
BDI: Beck Depression Inventory
BDI-1A: Beck Depression Inventory-1A
BDTD: Brazilian Digital Library of Theses and Dissertations
BIC: Behavioral Insomnia of Childhood
BIS: Bergen Insomnia Scale
BMI: Body Mass Index
C1/C2: Cohort 1/Cohort 2
CES-D: Centre for Epidemiological Studies Depression Scale
CI: Chronic Insomnia
COMISA: Comorbid Insomnia and Sleep Apnoea
CPC: Cardiopulmonary Coupling
DeCS: Health Sciences Descriptors
DIMS-F: Difficulty Initiating/Maintaining Sleep in the presence of daytime Fatigue
DSM-IV: Diagnostic and Statistical Manual of Mental Disorders-Fourth Edition
ECG: Electrocardiogram
EEG: Electroencephalogram
ESS: Epworth Sleepiness Scale
F: Female
FQ: Fatigue Questionnaire
GAD-7: Generalized Anxiety Disorder Screener
GER: Gastroesophageal Reflux
GMV: Gray Matter Volume
GRADE: Grading of Recommendations Assessment, Development and Evaluation
HAMA: Hamilton Anxiety Scale
HAMD: Hamilton Depression Scale
ICSD-2/ ICSD-3: International Classification of Sleep Disorders
IGI/ISI: Insomnia Severity Index
INS: Insomnia
ISI: Insomnia Severity Index
M: Male
MeSH: Medical Subject Headings
MFI: Multidimensional Fatigue Inventory
NC: Normal Controls
ODI: Oxygen Desaturation Index
OR: Odds Ratio
OSA: Obstructive Sleep Apnoea
OSA/OSA-I: Obstructive Sleep Apnoea / Obstructive Sleep Apnoea and Insomnia
OSA: Obstructive Sleep Apnoea Syndrome
PCL-5: PTSD Checklist for DSM-5
PHQ-9: Patient Health Questionnaire-9
PPG: Photoplethysmography
PPI: Psychophysiological Insomnia
PRISMA: Preferred Reporting Items for Systematic Reviews and Meta-Analyses
PSG: Polysomnography
PSQI: Pittsburgh Sleep Quality Index
REI: Respiratory Event Index
REM: Rapid Eye Movement
RL: Restless Legs
SA: Sleep Apnoea
SAI: Sleep Apnoea Indicator
SD: Standard Deviation
SDB: Sleep-Disordered Breathing
SE: Sleep Efficiency
SOL: Sleep Onset Latency
SpO2: Peripheral Oxygen Saturation
SQI: Sleep Quality Index
SSM: Sleep State Misperception
Structural MRI: Structural Magnetic Resonance Imaging
TST: Total Sleep Time
WASO: Wake Time After Sleep Onset
Wrist-PPG: Photoplethysmography/accelerometry
