# Supplementary material for: Physiopathological correlations of comorbid insomnia and sleep apnoea (comisa) – a systematic review and meta-analysis
Source: Sleep Breath. 2026 Mar 21;30(2):101. doi: 10.1007/s11325-026-03631-0 (PMC13005779; doi:10.1007/s11325-026-03631-0)
Supplement: Supplementary file 1 — Supplementary Material 1 (DOCX 1.73 MB) [file 11325_2026_3631_MOESM1_ESM.docx]

**PHYSIOPATHOLOGICAL CORRELATIONS OF COMORBID INSOMNIA AND SLEEP APNEA (COMISA) – A SYSTEMATIC REVIEW AND**

**META-ANALYSIS**

[**Sleep and Breathing**](https://link.springer.com/journal/11325)

**International Journal of the Science and Practice of Sleep Medicine**

**Springer Signature**

Ervin Cotrik (Postgraduate Program in Medical Sciences; Sleep Disorders Service of the Divisionof Otolaryngology, Head and Neck), University of Campinas - UNICAMP, Brazil (corresponding author).

Dr. Janete Hernandes, Instituto de Pesquisa Capel Castro (Department of Sleep Medicine Research), Goiânia, Goiás, Brasil.

Dr. Viviane Castro, Instituto de Pesquisa Capel Castro (Department of Sleep Medicine Research), Goiânia, Goiás, Brasil.

Dr. Edilson Zancanella, UNICAMP (Sleep Disorders Service of the Division of Otolaryngology, Head and Neck), Campinas, São Paulo, Brasil.

**Correspondent author’s email:** [cotrikpsiquiatria@gmail.com](mailto:cotrikpsiquiatria@gmail.com)

Supplementary Material  **1**. Study Search results

| **Databasis** | **Search strategies** | **Results** |
| --- | --- | --- |
| PubMed | Search: (((COMISA[Title/Abstrac t]) OR (Co-morbid insomnia[Title/Abstract] AND sleep apnoea[Title/Abstract])) OR (Comorbid insomnia[Title/Abstract] AND sleep apnoea[Title/Abstract])) AND (pathophysiology) | 10 |
| Embase | comisa OR ('co-morbid insomnia':ti,ab,kw AND 'sleep apnoea':ti,ab,kw) OR ('comorbid insomnia':ti,ab,kw AND 'sleep apnoea':ti,ab,kw) AND pathophysiology | 12 |
| Scopus | ( ALL ( pathophysiology ) ) AND ( ( TITLE-ABS-KEY ( comisa ) OR TITLE-ABS-KEY (  co-morbid AND insomnia AND sleep AND apnoea ) OR  TITLE-ABS-KEY ( comorbid AND insomnia AND sleep AND apnoea ) ) ) | 148 |
| Web of science | COMISA (Topic) or Co-morbid insomnia and sleep apnoea (Topic) or Comorbid insomnia and sleep apnoea (Topic) AND pathophysiology (All Fields) | 20 |
| Cochrane Library | (COMISA):ti,ab,kw OR  (Co-morbid insomnia and sleep | 06 |

apnoea):ti,ab,kw OR (Comorbid insomnia and sleep apnoea):ti,ab,kw AND (pathophysiology)

| BDTD (Biblioteca Digital Brasileira de Teses e Dissertações) | (Todos os campos: COMISA) | 01 |
| --- | --- | --- |
| https://researchrabbitapp.com/ | Comorbid Insomnia and Sleep Apnea, COMISA | 52 |
| **Total** |  | 249 |
