# Supplementary material for: Physiopathological correlations of comorbid insomnia and sleep apnoea (comisa) – a systematic review and meta-analysis
Source: Sleep Breath. 2026 Mar 21;30(2):101. doi: 10.1007/s11325-026-03631-0 (PMC13005779; doi:10.1007/s11325-026-03631-0)
Supplement: Supplementary file 2 — Supplementary Material 2 (DOCX 2.58 MB) [file 11325_2026_3631_MOESM2_ESM.docx]

**PHYSIOPATHOLOGICAL CORRELATIONS OF COMORBID INSOMNIA AND SLEEP APNEA (COMISA) – A SYSTEMATIC REVIEW AND**

**META-ANALYSIS**

[**Sleep and Breathing**](https://link.springer.com/journal/11325)

**International Journal of the Science and Practice of Sleep Medicine**

**Springer Signature**

Ervin Cotrik (Postgraduate Program in Medical Sciences; Sleep Disorders Service of the Divisionof Otolaryngology, Head and Neck), University of Campinas - UNICAMP, Brazil (corresponding author).

Dr. Janete Hernandes, Instituto de Pesquisa Capel Castro (Department of Sleep Medicine Research), Goiânia, Goiás, Brasil.

Dr. Viviane Castro, Instituto de Pesquisa Capel Castro (Department of Sleep Medicine Research), Goiânia, Goiás, Brasil.

Dr. Edilson Zancanella, UNICAMP (Sleep Disorders Service of the Division of Otolaryngology, Head and Neck), Campinas, São Paulo, Brasil.

**Correspondent author’s email:** [cotrikpsiquiatria@gmail.com](mailto:cotrikpsiquiatria@gmail.com)

Supplementary Material 2.. Description of the selected studies on COMISA

| Source | Study type | Country of Study | Study Objectives | Sample size | Sample characteristics | Criteria for COMISA diagnosis | COMISA prevalence | Study conclusions | Risk of bias |
| --- | --- | --- | --- | --- | --- | --- | --- | --- | --- |
| Cruz et al., 2022 | Cross-sectional | Portugal, Brazil, USA | Assess the frequency of co-occurrence of sleep-related respiratory disorders and insomnia in children and adolescents. | Coorte 1: 50; Coorte 2: 384  participants | Students (children/adolescents); Average Age: 13.6 (C1) / 12.9 (C2); Sex: 58% M (C1) / 55% M (C2); BMI: 23.0 (C1) / 26.0 (C2); | High risk of OSA (≥3 affirmative responses to risk questions) and insomnia. For Cohort 2: SDB (AHI >2 events/h) and OSA (obstructive AHI >5 events/h). | 16%–18%. | In both cohorts, COMISA manifested as a greater tendency toward sleepiness and fatigue. Thus, the presence of COMISA is common in the pediatric age group and is accompanied by a more prominent symptomatic phenotype | Moderate |
| Khazaie et al., 2024 | Cross-sectional | Iran | Investigate the prevalence and clinical associations of COMISA in a large sleep clinic sample. | 1807 participants | Patients; Average Age: 49.26; Sex: 61.2% Male / 38.8% Female; Average BMI: 31.11; | SA (AHI ≥5) and INS (chronically confirmed by psychiatrists). COMISA: both present. | 26.6%. | COMISA is prevalent in sleep clinics in Iran. Male gender and advanced age are significantly associated with COMISA | Low |
| Kundu, et al., 2022 | Cross-sectional | Índia | Determine the proportion of SSM and COMISA in patients with OSA and chronic insomnia. Compare sleep macroarchitecture. | 32  participants | Patients; Average Age: ~50; Sex: 75% Male (Insomnia) / 81.25% Female (OSA); Average BMI: 26.98 (Insomnia) / 31.33 (OSA); | Chronic insomnia (ICSD-3 criteria) and OSA (AHI >5). | **18%** - **43%**. | SSM is common among individuals with OSA and chronic insomnia. COMISA was more frequent among patients with OSA compared to those with insomnia. Sleep macroarchitecture is comparable across groups. Identifying COMISA is relevant for optimal management. | Moderate |
| Luciano, 2024 | Coorte | Brazil (São Paulo) | Investigate the prevalence and incidence of COMISA in the population of São Paulo (EPISONO 2007 and 2015). | 585 participants | General population (EPISONO); Age: 20 to 80 years (range); Sex: Mixed population; BMI: Not specified; | Insomnia (ISI: 8–28) and OSA (AHI: ≥5 with symptoms / ≥15 without symptoms). | **2007:** **17.64%.** **2015:** **22.95%.** | The prevalence of COMISA is high and shows a significant increase over an 8-year follow-up. The incidence of COMISA is also high, affecting approximately one-sixth of individuals who did not present this condition at the initial assessment. The presence of one disorder increases the likelihood of developing the other. | Low |
| Misliviec et al., 2022 | Cross-sectional | USA | Characterize insomnia, OSA, and COMISA in active-duty military personnel. Determine differences in symptoms | 309  participants | Active duty military personnel; Average age: 37.17; Sex: 62.1% male / 37.9% female; Average BMI: 28.73; | ICSD-3 criteria for chronic insomnia and OSA (AHI ≥5). Insomnia symptoms not exclusively attributable to disordered breathing. | **36.9%.** | Military personnel with insomnia and COMISA had greater morbidity than those with OSA alone. The high prevalence of sleep disorders associated with mental disorders underscores the importance of developing specific treatments. Higher prevalence of depression and anxiety in the insomnia/COMISA groups. | Low |
| Pan et al., 2024 | Control Case | China | Investigate changes in gray matter volume (GMV) in patients with COMISA and its relationship with clinical measures. | 58  participants | Patients; Average Age: 41.91; Sex: 30 Male / 4 Female; Average BMI: 29.6; | ICSD-3 criteria for OSA and chronic insomnia (≥3 times/week for >3 months). Subdivided by OSA severity (AHI 5–30; AHI >30). | **100%.** | Findings showed an expansion of GMV atrophy from the temporal lobe to the limbic system (right amygdala) as severity stages increased in patients with COMISA. | Moderate |
| Paramo-Brando et al., 2019 | Cross-sectional | Mexico | Compare EEG spectra in COMISA and sleep apnea only during pre-sleep wakefulness, N1, and REM. | 20 participants | Patients; Average Age: 38.7 (COMISA) / 41.6 (OSA); Sex: 70% M (COMISA) / 60% F (OSA); Average BMI: 26.0 (COMISA) / 28.1 (OSA); | OSAS (AHI >5). COMISA: additionally, difficulties initiating/maintaining sleep, non-restorative sleep for ≥1 month, and associated impairment. | Not applicable (selected sample). | Patients with COMISA, compared to those with OSA alone, exhibited higher left high-frequency rhythms during pre-sleep wakefulness and REM sleep, which may be due to increased emotional and cognitive activity. During stage N1, they showed greater left delta power, suggesting some slowing after sleep deprivation. | Moderate |
| Subramanian et al., 2021 | Prevalence | EUA | Describe the prevalence of COMISA in a population with sleep apnea and characterize its features. | 296 participants | Patients; Median age: 50; Sex: 49% male / 51% female; Median BMI: 41.5; | OSA (AHI ≥5) and ≥2 main insomnia symptoms (difficulty falling asleep, staying asleep, or early awakening). | **63%.** | Insomnia is extremely prevalent in patients with OSA, associated with daytime sleepiness, PPI symptoms (psychophysiological insomnia), GER (gastroesophageal reflux), and RL (restless legs). | Low |
| Wu et al., 2024 | Cross-sectional | China | Investigate the prevalence of COMISA related to age. | 2176 participants | Community/referenced cohort; Average age: 60.3; Sex: 52% male / 48% female; BMI: Not reported; | AHI ≥5 and presence of (clinical) insomnia. | **45.1%.** | Significant association between increasing age and the development of COMISA. COMISA is more frequent in women, and patients have longer sleep onset latency, shorter total sleep time, and lower AHI.. | Moderate |
| Wulterkens et al., 2024 | Longitudinal | Netherlands | Explore sleep architecture in patients with OSA and COMISA using single-night PSG and multi-night PPG/actigraphy. | 117  participants | Patients with suspected OSA; Median Age: 51; Sex: 65.7% M / 34.3% F; Median BMI: 28.3; | OSA (ICSD-3, AHI ≥5). Clinically significant insomnia (ISI sub-score ≥4 + complaints). COMISA: both. | **57,2%.** | Single-night PSG showed no significant differences. Multi-night measurements: COMISA had longer sleep onset latency, lower sleep efficiency, longer and more variable awakenings. | Low |
| Yelov et al., 2024 | Cross-sectional | Israel | Examine the association between Pediatric Behavioral Insomnia (BIC) and OSA in young children. | 312  participants | Children; Average Age: 4.42; Sex: 62.5% Male / 37.5% Female; BMI: 45.79 (percentile); | Pediatric Behavioral Insomnia (clinical, ICSD-3) and OSA (AHI >2). COMISA: both present. | **8.7%.** | There is no association between Pediatric Behavioral Insomnia and OSA in children. Children with COMISA were significantly younger. Healthcare professionals should consider both disorders separately | Moderate |
| Bjorvatn et al., 2014 | Cross-sectional | Norway | Investigate the prevalence and correlates of insomnia and excessive sleepiness/hypersomnia in participants with OSA symptoms. | 1502 participants | General population; Average age: 53.6; Sex: 50.7% male / 49.3% female; BMI: Not reported; | Insomnia (≥3 days/week in sleep and daytime items) and OSA symptoms (self-reported) | **3.5%.** | Insomnia twice as common (and hypersomnia three times more) among participants with OSA symptoms. Non-restorative sleep is the most common complaint (55%). | Moderate |
| Choi et al., 2020 | Cross-sectional | Korea | Investigate differences in sleep perception in chronic insomnia (CI), COMISA (OSA-I), OSA only, and normal controls (NC). | 360  participants | Patients; Median age: ~49-61; Sex: Predominantly male; Median BMI: ~23-25.5; | CI (chronic insomnia, AHI <5), OSA-I (OSA AHI ≥5 + insomnia complaints), OSA (OSA only), NC (healthy AHI <5). | **13.6%.** | Patients with COMISA reported the smallest discrepancy between habitual sleep duration and subjective total sleep time. PSG profiles are distinct for OSA with or without insomnia. | Moderate |
| Hilmisson et al. 2019 | Cross-sectional | Iceland | Determine the prevalence of undiagnosed SDB in patients with chronic insomnia. | 110 participants | Patients with chronic insomnia undergoing pharmacological treatment; Average Age: 49.9; Sex: 12% Male / 88% Female; Average BMI: 32.0; | Chronic insomnia and undiagnosed SDB/OSA (Respiratory Event Index – REI >15). | **25%.** | High prevalence and symptom overlap justify objective testing before therapy. Diagnostic caution for women with insomnia, as SDB may not be considered. | Low |
| Lang et al., 2017 | Prevalance | Australia | Examine the prevalence and profile of undiagnosed COMISA in men in the community. | 700  participants | Community; Average Age: 58.5; Sex: 100% Male; Average BMI: 29.1; | Insomnia: DIMS-F (difficulty initiating/maintaining sleep in the presence of daytime fatigue). OSA: AHI ≥10. COMISA: both conditions | **6.7%.** | Men with COMISA have higher prevalence and severity of depression compared to men with only one disorder.. | Low |
| Wulterkens et al., 2023 | Cross-sectional | Netherlands | Investigate differences in sleep architecture among patients with OSA, insomnia, and COMISA to improve COMISA diagnosis. | 326  participants | Patients; Median Age: 56.0; Sex: 66.6% M / 33.4% M; Median BMI: 27.9 (OSA) / 27.5 (COMISA); | OSA and insomnia diagnosed by ICSD-2/ICSD-3. Insomnia: difficulties initiating/maintaining sleep + daytime consequences (≥3 months, ≥3 times/week). COMISA: both conditions.. | **24.5%.** | COMISA shows prolonged awakenings. Wake after sleep onset and long awakenings significantly higher in COMISA/Insomnia vs OSA.Total sleep time and sleep efficiency higher in OSA vs COMISA/Insomnia.COMISA results in greater sleep disruption. | Low |

Legend: AHI (Apnea-Hypopnea Index); BIC (Behavioral Insomnia of Childhood); BMI (Body Mass Index); C1 / C2 (Cohort 1 / Cohort 2); CI (Chronic Insomnia); COMISA (Comorbid Insomnia and Sleep Apnea); DIMS-F (Difficulty Initiating/Maintaining Sleep in the presence of daytime Fatigue); EEG (Electroencephalogram); ESS (Epworth Sleepiness Scale); F (Female); GER (Gastroesophageal Reflux); GMV (Gray Matter Volume); ICSD-2 / ICSD-3 (International Classification of Sleep Disorders); IGI / ISI (Insomnia Severity Index); INS (Insomnia); M (Male); NC (Normal Controls); OSA / OSA-I (Obstructive Sleep Apnea / Obstructive Sleep Apnea and Insomnia); OSAS (Obstructive Sleep Apnea Syndrome); PPG (Photoplethysmography); PPI (Psychophysiological Insomnia); PSG (Polysomnography); REI (Respiratory Event Index); RL (Restless Legs); SA (Sleep Apnea); SDB (Sleep-Disordered Breathing); SSM (Sleep State Misperception); TST (Total Sleep Time).
