# Supplementary material for: Physiopathological correlations of comorbid insomnia and sleep apnoea (comisa) – a systematic review and meta-analysis
Source: Sleep Breath. 2026 Mar 21;30(2):101. doi: 10.1007/s11325-026-03631-0 (PMC13005779; doi:10.1007/s11325-026-03631-0)
Supplement: Supplementary file 3 — Supplementary Material 3 (DOCX 1.74 MB) [file 11325_2026_3631_MOESM3_ESM.docx]

**PHYSIOPATHOLOGICAL CORRELATIONS OF COMORBID INSOMNIA AND SLEEP APNEA (COMISA) – A SYSTEMATIC REVIEW AND**

**META-ANALYSIS**

[**Sleep and Breathing**](https://link.springer.com/journal/11325)

**International Journal of the Science and Practice of Sleep Medicine**

**Springer Signature**

Ervin Cotrik (Postgraduate Program in Medical Sciences; Sleep Disorders Service of the Divisionof Otolaryngology, Head and Neck), University of Campinas - UNICAMP, Brazil (corresponding author).

Dr. Janete Hernandes, Instituto de Pesquisa Capel Castro (Department of Sleep Medicine Research), Goiânia, Goiás, Brasil.

Dr. Viviane Castro, Instituto de Pesquisa Capel Castro (Department of Sleep Medicine Research), Goiânia, Goiás, Brasil.

Dr. Edilson Zancanella, UNICAMP (Sleep Disorders Service of the Division of Otolaryngology, Head and Neck), Campinas, São Paulo, Brasil.

**Correspondent author’s email:** [cotrikpsiquiatria@gmail.com](mailto:cotrikpsiquiatria@gmail.com)

Supplementary Material 3. Instruments for the assessment of Obstructive Sleep Apnea and Insomnia (COMISA), Sleepiness, Fatigue, Mood Disorders, Depression and Anxiety, more detailed evaluation of sleep, breathing, and gray matter analysis.

| **Clinical condition assessed** | **Instruments** | **Authors** |
| --- | --- | --- |
|  | Polysomnography (PSG) | Cruz (2022), Khazaie (2024), Kundu e Luciano (2024), Misliviec (2022), Pan (2024),  Paramo (2019), Subramanian  (2021), Wu (2024), Wulterkens (2024), Yelov e Choi (2020), Lang (2017), Wulterkens et al. (2023). |
| Obstructive Sleep Apnea (OSA) |  |  |
|  | Apnea-Hypopnea Index (AHI) | Cruz (2022), Khazaie (2024),  Kundu e Luciano (2024), Misliviec (2022), Pan (2024),  Paramo (2019), Subramanian  (2021), Wu (2024), Wulterkens (2024), Yelov e Choi (2020), Lang (2017), Wulterkens et al. (2023). |
|  | Respiratory Event Index (REI) | Hilmisson (2019). |



|  | Oxygen Desaturation Index (ODI) | Wulterkens (2024) e Lang (2017). |
| --- | --- | --- |
|  | STOP-Bang and Berlin questionnaires | Cruz (2022), Luciano (2024) e  Hilmisson (2019). |
|  | Insomnia Severity Index (ISI) | Luciano (2024), Misliviec  (2022), Pan (2024), Wulterkens  (2024) e Hilmisson (2019). |
|  | Bergen Insomnia Scale (BIS) | Bjorvatn (2014). |
|  | Pittsburgh Sleep Quality Index (PSQI) | Misliviec (2022), Pan (2024) e  Lang (2017). |
| Insomnia | Clinical interviews and self-reports. | Khazaie (2024), Kundu e Pan (2024), Paramo (2019), Wu  (2024), Wulterkens (2024), Yelov e Bjorvatn (2014), Choi (2016), Wulterkens et al. (2023). |
| Sleepiness | Epworth Sleepiness Scale (ESS) | Cruz (2022), Khazaie (2024), Kundu e Luciano (2024), Misliviec (2022), Pan (2024), Subramanian (2021), Yelov e Bjorvatn (2014), Choi (2020),  Hilmisson (2019), Lang (2017). |
|  | PTSD Checklist for DSM-5 (PCL-5), Generalized Anxiety Disorder Screener (GAD-7), Patient Health Questionnaire-9 **(**PHQ-9) | Misliviec (2022). |
| Mood disorders, depression and anxiety | HAMA: Hamilton Anxiety Scale, HAMD: Hamilton Depression Scale | Pan (2024). |
|  | Beck Depression Inventory (BDI) | Choi (2020). |
|  | Centre for Epidemiological Studies Depression Scale **(**CES-D), Beck Depression Inventory-1A **(**BDI-1A), Patient Health Questionnaire-9 **(**PHQ-9) | Lang (2017). |
|  | Chalder Fatigue Scale | Luciano (2024). |
| Fatigue. | Multidimensional Fatigue Inventory **(**MFI) | Misliviec (2022). |
|  | Fatigue Questionnaire (FQ) | Bjorvatn (2014). |
|  | Electroencephalography (EEG) | Paramo (2019) |

| More detailed evaluation of sleep and breathing. | Photoplethysmography/accelero metry (wrist-PPG). | Wulterkens (2024) |
| --- | --- | --- |
|  | ECG via Cardiopulmonary Coupling (CPC) with Sleep Quality Index (SQI) | Hilmisson (2019) |
|  | Sleep Apnea Indicator (SAI) | Hilmisson (2019). |
| Gray matter analysis | Structural magnetic resonance imaging (structural MRI) | Pan (2024) |



Legend: AHI, Apnea-Hypopnea Index, BDI, Beck Depression Inventory, BDI-1A, Beck Depression Inventory-1A, BIS, Bergen Insomnia Scale, CES-D, Centre for Epidemiological Studies Depression Scale, COMISA, Comorbid Insomnia and Sleep Apnea, CPC, Cardiopulmonary Coupling, ECG, Electrocardiogram, EEG, Electroencephalography, ESS, Epworth Sleepiness Scale, FQ, Fatigue Questionnaire, GAD-7, Generalized Anxiety Disorder Screener, HAMA, Hamilton Anxiety Scale, HAMD, Hamilton Depression Scale, ISI, Insomnia Severity Index, MFI, Multidimensional Fatigue Inventory, ODI, Oxygen Desaturation Index, OSA, Obstructive Sleep Apnea, PCL-5, PTSD Checklist for DSM-5, PHQ-9, Patient Health Questionnaire-9, PSG, Polysomnography, PSQI, Pittsburgh Sleep Quality Index, REI, Respiratory Event Index, SAI, Sleep Apnea Indicator, SQI, Sleep Quality Index, structural MRI, Structural Magnetic Resonance Imaging, wrist-PPG, Photoplethysmography/accelerometry.
