# Supplementary material for: Physiopathological correlations of comorbid insomnia and sleep apnoea (comisa) – a systematic review and meta-analysis
Source: Sleep Breath. 2026 Mar 21;30(2):101. doi: 10.1007/s11325-026-03631-0 (PMC13005779; doi:10.1007/s11325-026-03631-0)
Supplement: Supplementary file 4 — Supplementary Material 4 (DOCX 1.74 MB) [file 11325_2026_3631_MOESM4_ESM.docx]

**PHYSIOPATHOLOGICAL CORRELATIONS OF COMORBID INSOMNIA AND SLEEP APNEA (COMISA) – A SYSTEMATIC REVIEW AND**

**META-ANALYSIS**

[**Sleep and Breathing**](https://link.springer.com/journal/11325)

**International Journal of the Science and Practice of Sleep Medicine**

**Springer Signature**

Ervin Cotrik (Postgraduate Program in Medical Sciences; Sleep Disorders Service of the Divisionof Otolaryngology, Head and Neck), University of Campinas - UNICAMP, Brazil (corresponding author).

Dr. Janete Hernandes, Instituto de Pesquisa Capel Castro (Department of Sleep Medicine Research), Goiânia, Goiás, Brasil.

Dr. Viviane Castro, Instituto de Pesquisa Capel Castro (Department of Sleep Medicine Research), Goiânia, Goiás, Brasil.

Dr. Edilson Zancanella, UNICAMP (Sleep Disorders Service of the Division of Otolaryngology, Head and Neck), Campinas, São Paulo, Brasil.

**Correspondent author’s email:** [cotrikpsiquiatria@gmail.com](mailto:cotrikpsiquiatria@gmail.com)

Supplementary Material 4. Risk of bias evaluation (Joana Briggs Institute Tools)

| Study/Questions | Q1 | Q2 | Q3 | Q4 | Q5 | Q6 | Q7 | Q8 | Q9 | Q10 | Q11 | % |
| --- | --- | --- | --- | --- | --- | --- | --- | --- | --- | --- | --- | --- |
| Wulterkens et al. (2023)  Cross-sectional study | Y | Y | Y | Y | Y | N | Y | Y | NA | NA | NA | 87% |
| Bjorvatn et al. (2014)  Cross-sectional study | Y | Y | Y | Y | N | N | N | Y | NA | NA | NA | 62% |
| Choi et al. (2020).  Cross-sectional study | Y | Y | Y | Y | N | N | Y | Y | NA | NA | NA | 75% |
| Hilmisson et al. (2019)  Cross-sectional study | Y | Y | Y | Y | Y | N | Y | Y | NA | NA | NA | 87% |
| Kundu et al. (2022)  Cross-sectional study | Y | Y | Y | Y | N | N | Y | Y | NA | NA | NA | 75% |
| Khazaie et al. (2024)  Cross-sectional study | Y | Y | Y | Y | Y | N | Y | Y | NA | NA | NA | 87% |
| Lang et al. (2017)  Prevalence study | Y | Y | NC | Y | Y | Y | Y | Y | Y | NA | NA | 89% |
| Luciano (2024)  Prevalence study | Y | Y | Y | Y | Y | Y | Y | Y | Y | NA | NA | 100% |
| Cruz et al. (2022).  Cross-sectional study | Y | Y | Y | N | N | N | Y | Y | NA | NA | NA | 62  % |
| Pan et al. (2024).  Caso controle | N | N | N | Y | Y | Y | Y | Y | Y | Y | NA | 70  % |
| Páramo-Brando et al. (2019)  Cross-sectional study | Y | Y | Y | Y | N | N | Y | Y | NA | NA | NA | 75  % |

| Subramanian et al. (2021).  Prevalence study | Y | Y | NC | Y | Y | Y | Y | Y | NC | NA | NA | 88  % |
| --- | --- | --- | --- | --- | --- | --- | --- | --- | --- | --- | --- | --- |
| Mysliwiec et al. (2022)  Cross-sectional study | Y | Y | Y | Y | Y | N | Y | Y | NA | NA | NA | 87  % |
| Wu et al. (2024)  Cross-sectional study | Y | Y | Y | Y | N | N | Y | Y | NA | NA | NA | 75  % |
| Wulterkens et al. (2024).  Longitudinal study | Y | Y | Y | Y | Y | NA | Y | Y | Y | NA | Y | 82  % |
| Yelov et al. (2024).  Cross-sectional study | Y | Y | Y | Y | Y | N | N | Y | Y | NA | NA | 78  % |

Legend: Y= Yes; N= No; NC= Not clear; NA= Not applicable; % Percentage of study quality
