# Supplementary material for: Physiopathological correlations of comorbid insomnia and sleep apnoea (comisa) – a systematic review and meta-analysis
Source: Sleep Breath. 2026 Mar 21;30(2):101. doi: 10.1007/s11325-026-03631-0 (PMC13005779; doi:10.1007/s11325-026-03631-0)
Supplement: Supplementary file 5 — Supplementary Material 5 (DOCX 1.74 MB) [file 11325_2026_3631_MOESM5_ESM.docx]

Physiopathological Correlations of Comorbid Insomnia and Sleep Apnea (COMISA) – A Systematic Review and Meta-Analysis

[**Sleep and Breathing**](https://link.springer.com/journal/11325)

**International Journal of the Science and Practice of Sleep Medicine**

**Springer Signature**

Ervin Cotrik (Postgraduate Program in Medical Sciences; Sleep Disorders Service of the Divisionof Otolaryngology, Head and Neck), University of Campinas - UNICAMP, Brazil (corresponding author).

Dr. Janete Hernandes, Instituto de Pesquisa Capel Castro (Department of Sleep Medicine Research), Goiânia, Goiás, Brasil.

Dr. Viviane Castro, Instituto de Pesquisa Capel Castro (Department of Sleep Medicine Research), Goiânia, Goiás, Brasil.

Dr. Edilson Zancanella, UNICAMP (Sleep Disorders Service of the Division of Otolaryngology, Head and Neck), Campinas, São Paulo, Brasil.

**Correspondent author’s email:** [cotrikpsiquiatria@gmail.com](mailto:cotrikpsiquiatria@gmail.com)

Supplementary Material 5. Standardization of results for mean and standard deviation.

| Authors | Patient Group | AHI  (Events  /hour). | Minim um SpO2 (%) | Micro-arousal Index (Events/hour) | Sleep Efficienc y (%) | Sleep Latency (minutes) | REM Sleep Duration (% or min). | Awakeni ng Index |
| --- | --- | --- | --- | --- | --- | --- | --- | --- |
| Khazaie et al. (2024) | COMISA | 33.90(±  34.62) | NI | 31.33(±26.86) | 67.0(±0.  22) | 15.06(±26.  90) | 15.47(±41.27) | 49.92(±  41.0) |
| Khazaie et al. (2024) | Isolated Obstructive Sleep Apnea | 37.79(±  31.16) | NI | 31.47(±25.93) | 71.0(±0.  20) | 13.49(±27.  22) | 11.49(±15.70) | 45.10(±  40.94) |
| Khazaie et al. (2024) | Isolated Insomnia | 1.97(±1  .47) | NI | 21.66(±17.54) | 72.0(±0.  19) | 23.30(±28.  72) | 19.05(±17.26) | 67.13(±  51.53) |
| Kundu (2021) | COMISA | 33.30(±  29.85) | NI | 13.90 (±  14.52) | 81.90 (±  12.44) | 12.00 (±  29.70) | 10.70 (± 5.26) | 8.00 (±  2.96) |
| Kundu (2021) | Isolated Obstructive Sleep Apnea | 46.60 (± 36.30) | NI | 28.75 (±  20.85) | 75.40 (±  12.44) | 13.80 (±  43.11) | 14.80 (± 1.85) | 12.00 (±  8.30) |
| Kundu (2021) | Isolated Insomnia | 6.00 (±  5.70) | NI | 12.30 (± 8.22) | 78.50 (±  6.74) | 35.70 (±  31.70) | 21.10 (± 4.59) | 5.50 (±  2.59) |
| Mysliwiec et al. (2022) | COMISA | 17.91(±  14.28) | 87.87(  ±4.84) | 24.58(±12.43) | 82.39(±1  1.73) | 20.24(±19.  81) | 18.17(±6.75) | 54.99(±  42.72) |
| Mysliwiec et al. (2022) | Isolated Obstructive Sleep Apnea | 23.13(±  21.05) | 87.27(  ±4.68) | 25.86(±16.93) | 85.64(±1  0.92) | 12.86(±14.  64) | 18.85(±6.21) | 48.43(±  39.72) |
| Mysliwiec | Isolated | 2.47(±1 | 92.23( | 14.88(±6.63) | 84.46(±9 | 20.75(±21. | 18.2(±7.21) | 45.2(±3 |

et al. (2022) Insomnia .33) ±2.51) .7) 82) 1.53)

| Páramo et al. (2019) | COMISA | 11.5(26  .7) | 86.5(7) | NI | 82.5(11.3  ) | 11.7(12.8) | 10.4(11.1) | 10.7(±1  2.5) |
| --- | --- | --- | --- | --- | --- | --- | --- | --- |
| Páramo et al. (2019) | Isolated Obstructive Sleep Apnea | 22.3(25  .8) | 83(12) | NI | 91.9(9.6) | 11.3(11.9) | 14.3(7.5) | 5.7(±26.  5) |
| Wu et al. (2024) | COMISA | 26.5(±2  0.5) | NI | NI | NI | 24.1(±30.2) | 15.6(±6.8) | 114(±77  ) |
| Wu et al. (2024) | Isolated Obstructive Sleep Apnea | 40.9(±2  3.9) | NI | NI | NI | 18.3(±27.6) | 16.4(±6.2) | 07(±72) |
| Wulterkens et al. (2024) | COMISA | 16.6(10  .1,29.4) | NI | NI | 80.8(±1.  06) | 17.1(±1.96) | 18.6(±0.83) | 33.1(±1.  56) |
| Wulterkens et al. (2024) | Isolated Obstructive Sleep Apnea | 22.2(11  .3,33.0) | NI | NI | 84.3(±1.  12) | 16.0(±2.13) | 20.1(±9.90) | 31.5(±1.  72) |
| Yelov et al. (2024) | COMISA | 9.85(±6  .98) | 82.30(  ±5.38) | 7.43(±4.74) | 78.64(±1  1.05) | 24.05(±23.  30) | 18.33(±4.92) | 63.13(±  44.61) |
| Yelov et al. (2024) | Isolated Obstructive Sleep Apnea | 10.88(±  12.15) | 81.84(  ±8.08) | 7.52(±4.67) | 81.48(±1  0.70) | 32.50(±31.  17) | 17.73(±5.57) | 51.27(±  38.43) |
| Yelov et al. (2024) | Isolated Insomnia | 0.82(±0  .60) | 89.90(  ±3.95) | 6.58(±2.61) | 80.20(±1  3.24) | 39.72(±38.  57) | 15.72(±5.43) | 44.47(±  52.04) |
| Choi et al. (2020) | COMISA | 9.30 (±  6.67) | NI | 23.50 (±  10.07) | 78.20 (±  17.19) | 15.50 (±  25.78) | 18.50 (± 6.52) | 18.00 (±  13.85) |
| Choi et al. (2020) | Isolated Obstructive Sleep Apnea | 21.00 (± 18.89) | NI | 28.10 (±  14.89) | 86.90 (±  6.74) | 5.00 (±  5.26) | 19.10 (± 5.85) | 11.80 (±  5.70) |
| Choi et al. (2020) | Isolated Insomnia | 0.60 (±  1.26) | NI | 17.40 (± 8.07) | 77.80 (±  12.74) | 12.60 (±  12.07) | 17.70 (± 7.33) | 18.00 (±  12.89) |
| Wulterkens et al. (2023) | COMISA | 16.60 (± 13.04) | NI | 0.50 (± 1.33) | 77.30 (±  9.56) | 12.50 (±  17.63) | 18.00 (± 6.07) | 36.00 (±  17.41) |
| Wulterkens et al. (2023) | Isolated Obstructive Sleep Apnea | 21.60 (± 17.26) | NI | 1.10 (± 2.74) | 84.40 (±  8.81) | 11.00 (±  12.44) | 18.50 (± 4.74) | 36.00 (±  16.67) |
| Wulterkens et al. (2023) | Isolated Insomnia | 6.80 (±  6.30) | NI | 0.00 (± 0.15) | 78.20(±1  3.78) | 12.50 (±  11.11) | 17.30 (± 4.59) | 32.00 (±  12.96) |
