# Supplementary material for: Physiopathological correlations of comorbid insomnia and sleep apnoea (comisa) – a systematic review and meta-analysis
Source: Sleep Breath. 2026 Mar 21;30(2):101. doi: 10.1007/s11325-026-03631-0 (PMC13005779; doi:10.1007/s11325-026-03631-0)
Supplement: Supplementary file 7 — Supplementary Material 7 (DOCX 2.61 MB) [file 11325_2026_3631_MOESM7_ESM.docx]

Physiopathological Correlations of Comorbid Insomnia and Sleep Apnea (COMISA) – A Systematic Review and Meta-Analysis

[**Sleep and Breathing**](https://link.springer.com/journal/11325)

**International Journal of the Science and Practice of Sleep Medicine**

**Springer Signature**

Ervin Cotrik (Postgraduate Program in Medical Sciences; Sleep Disorders Service of the Divisionof Otolaryngology, Head and Neck), University of Campinas - UNICAMP, Brazil (corresponding author).

Dr. Janete Hernandes, Instituto de Pesquisa Capel Castro (Department of Sleep Medicine Research), Goiânia, Goiás, Brasil.

Dr. Viviane Castro, Instituto de Pesquisa Capel Castro (Department of Sleep Medicine Research), Goiânia, Goiás, Brasil.

Dr. Edilson Zancanella, UNICAMP (Sleep Disorders Service of the Division of Otolaryngology, Head and Neck), Campinas, São Paulo, Brasil.

**Correspondent author’s email:** [cotrikpsiquiatria@gmail.com](mailto:cotrikpsiquiatria@gmail.com)

Supplementary Material 7. GRADE: COMISA and Isolated Insomnia



**Certainty assessment**

| Participants (Studies) | Risk of Bias | Inconsistency | Indirect Evidence | Imprecision | Other considerations | Overall certainty of evidence |
| --- | --- | --- | --- | --- | --- | --- |

**Apnea and Hypoapnea Index (AHI)**

|  | not | severe ^a^ | not | not severe | Publication bias | ⨁⨁⨁⨁ |
| --- | --- | --- | --- | --- | --- | --- |
| (6 observational | sever |  | severe |  | not detected. Has | High^a^ |
| studies) | e |  |  |  | a large magnitude |  |
|  | | | | | effect. |  |
|  |  |  |  |  | Potential |  |
|  |  |  |  |  | confounding |  |
|  |  |  |  |  | factors would |  |
|  |  |  |  |  | reduce the |  |
|  |  |  |  |  | demonstrated |  |
|  |  |  |  |  | effect. |  |
|  |  |  |  |  | Has a |  |
|  |  |  |  |  | dose-response |  |
|  |  |  |  |  | gradient. |  |

**SpO2 Mínimal**

(2 observational studies)

not sever e

severe ^b^ not severe

severe ^c^ Publication bias

not detected. Does not have a large magnitude effect.

Potential confounding factors would reduce the demonstrated effect.

Has a dose-response

gradient.

⨁⨁◯◯

_Low_b,c

| **Microarousal Index** | | | | | | |
| --- | --- | --- | --- | --- | --- | --- |
| (5 observational studies) | not sever e | severe ^d^ | not severe | not severe | Publication bias not detected. Does not have a large magnitude effect.  Potential confounding factors would reduce the demonstrated effect.  Has a dose-response  gradient. | ⨁⨁⨁◯  Moderate^d^ |
| **Arousal Index** | | | | | | |
| (6 observational studies) | not sever e | severe ^e^ | not severe | severe ^f^ | Publication bias not detected. Does not have a large magnitude effect.  Potential confounding factors would reduce the demonstrated effect.  Has a dose-response  gradient. | ⨁⨁◯◯  _Low_e,f |
| **Sleep Efficiency** | | | | | | |
| (6 observational studies) | not sever e | severe ^g^ | not severe | severe ^h^ | Publication bias not detected. Does not have a large magnitude effect.  Potential confounding factors would reduce the demonstrated effect.  Has a dose-response  gradient. | ⨁⨁◯◯  _Low_g,h |
| **Sleep Latency** | | | | | | |
| (6 observational studies) | not sever e | severe ^i^ | not severe | severe ^j^ | Publication bias not detected. Does not have a large magnitude effect.  Potential confounding factors would reduce the demonstrated effect.  Has a | ⨁⨁◯◯  _Low_i,j |

dose-response gradient.

| **REM Sleep Duration** | | | | | | |
| --- | --- | --- | --- | --- | --- | --- |
| (5 observational studies) | not sever e | not severe | not severe | severe ^k^ | Publication bias not detected. Does not have a large magnitude effect.  Potential confounding factors would reduce the demonstrated effect.  Has a dose-response  gradient. | ⨁⨁⨁◯  Moderate^k^ |

a. Statistically significant heterogeneity (p<0.05) and I² value (97%). b. Statistically significant heterogeneity (p<0.05) and I² value (83%). c. Sample size less than 400. d. Statistically significant heterogeneity (p<0.05) and I² value (95%). e. Statistically significant heterogeneity (p<0.05) and I² value (85%). f. The 95% CI crosses the central line. g. Statistically significant heterogeneity (p<0.05) and I² value (69%). h. The 95% CI crosses the central line. i. Statistically significant heterogeneity (p<0.05) and I² value (68%). j. The 95% CI crosses the central line. k. The 95% CI crosses the central line.
